# Integrative Multiscale Analysis Reveals EFNA1‐Driven Immune Remodeling Promotes Colorectal Cancer Lymph Node Metastasis

**DOI:** 10.1155/humu/8553028

**Published:** 2026-01-14

**Authors:** Wu Ning, Nan Qiao, Lei Zhou, Zongze Li, Lin Zhang, Xin Song

**Affiliations:** ^1^ Department of General Surgery, China–Japan Friendship Hospital, Beijing, China, zryhyy.com.cn

**Keywords:** CRC, EFNA1, lymphatic spread, single-cell sequencing, tumor ecosystem

## Abstract

**Background:**

Nodal involvement constitutes a pivotal prognostic indicator in colorectal carcinoma, yet the transcriptional machinery governing lymphatic dissemination and tumor‐microenvironment crosstalk remains poorly elucidated. Conventional bulk sequencing methodologies lack sufficient resolution to deconvolve functionally distinct malignant subclones that drive the metastatic cascade.

**Methods:**

We employed an integrative analytical framework combining tissue‐level gene expression profiling from TCGA and GEO repositories with eight single‐cell transcriptomic datasets comprising 266,995 individual cells. A phenotype‐guided computational algorithm was implemented to delineate metastasis‐driving malignant populations through correlating clinical parameters with cellular transcriptional profiles. Gene regulatory networks and transcription factor activity inference systematically decoded the molecular programs underlying metastatic phenotypes. Ligand‐receptor pairing analysis mapped intercellular communication architectures between neoplastic cells and microenvironmental constituents. Experimental validation encompassed genetic perturbation studies, functional characterization assays, and pharmacological response evaluation in preclinical systems.

**Results:**

We discovered a phenotypically distinct malignant population exhibiting robust associations with lymph node involvement and adverse clinical outcomes across nine independent validation cohorts. Regulatory network dissection identified IRF9 as the master transcriptional orchestrator of this metastatic program through coordination of a discrete gene module. Relative to their nonmetastatic counterparts, these aggressive cells establish markedly expanded intercellular communication networks, characterized by prominent VEGF‐driven angiogenic signaling to endothelial compartments and integrin‐laminin–mediated adhesion with stromal elements. EFNA1 emerged as a key signaling mediator demonstrating preferential enrichment in this metastatic subset. Elevated EFNA1 levels correlated with progressive disease stages and microsatellite‐stable subtypes while showing inverse relationships with PD‐L1 expression and T cell infiltration density—suggesting a unique immunoevasion mechanism. Genetic ablation of Efna1 substantially impaired cellular proliferation, motility, and invasion capabilities, while simultaneously augmenting Linifanib sensitivity, indicating synergistic therapeutic potential.

**Conclusions:**

Our investigation reveals a transcriptionally defined malignant population under IRF9 control that orchestrates immunosuppressive microenvironmental reprogramming via EFNA1‐mediated signaling networks. The EFNA1–Linifanib combination may represent a potential therapeutic approach to mitigate anti‐angiogenic resistance and restrain metastatic progression in colorectal carcinoma.

## 1. Introduction

Globally, colorectal cancer ranks as the third most common malignancy and the second leading cause of cancer mortality [1]. Lymph node and liver metastases represent two principal dissemination routes in this disease, both critically influencing therapeutic strategies and clinical prognosis. Within contemporary staging frameworks, nodal involvement status—determined through histopathological evaluation of surgically excised specimens—serves as a fundamental criterion for guiding postoperative adjuvant chemotherapy decisions [2]. Notwithstanding considerable progress in both operative interventions and multimodal therapeutic regimens, patients harboring lymph node involvement demonstrate substantially diminished cancer‐related survival rates [3], underscoring the imperative to elucidate the molecular machinery driving this metastatic process.

Contemporary investigations have demonstrated that lymphatic dissemination in colorectal malignancies follows intricate and heterogeneous patterns, characterized by tumor cell migration through diverse pathways within the lymphovascular architecture [4]. Although the precise mechanistic cascade remains incompletely delineated, prevailing theories posit that neoplastic cells migrate from the primary lesion to regional nodal basins via lymphatic channels before eventual systemic dissemination [1]. Conventional transcriptomic profiling at the tissue level, despite yielding important biological insights, inherently averages cellular signals and consequently obscures the heterogeneous composition of metastatic lesions and their associated microenvironments. High‐resolution characterization of tumor‐infiltrating immune populations has become increasingly recognized as essential for identifying modulators of disease progression and optimizing immunotherapeutic efficacy [5].

The emergence of single‐cell transcriptomic technologies has fundamentally transformed our capacity to interrogate tumor biology through unprecedented cellular resolution. Within neoplastic tissues, the immune compartment encompasses diverse cellular populations and molecular mediators that dynamically evolve during malignant progression, with bidirectional signaling between malignant and immune elements enabling malignant cells (MCs) to circumvent immunological detection and promote metastatic colonization [6]. Single‐cell resolution profiling enables comprehensive characterization of transcriptional heterogeneity and reveals cell‐type–specific mechanisms underlying immune evasion across different anatomical compartments [7]. This technology provides unique opportunities to dissect the cellular complexities inherent to lymph node metastases and identify immunomodulatory pathways operative during early‐stage dissemination [8]. Nevertheless, a significant methodological constraint persists: single‐cell datasets frequently lack comprehensive clinical annotation, and malignant populations within metastatic specimens exhibit functional heterogeneity regarding their metastatic competence, thereby complicating the precise delineation of metastasis‐driving cellular subsets.

Recent computational innovations have addressed this challenge by enabling integrated analysis of tissue‐level and single‐cell transcriptomic datasets, effectively bridging macroscopic phenotypic information with microscopic cellular heterogeneity. Analytical strategies combining both data modalities have demonstrated utility in identifying cancer‐associated markers at subpopulation resolution and constructing clinically relevant prognostic models [9]. These integrative frameworks exploit the rich clinical metadata associated with bulk profiling cohorts while simultaneously leveraging the granular cellular information inherent to single‐cell technologies, thereby facilitating identification of disease‐relevant cell states and their association with clinical trajectories. Prior investigations have successfully employed such approaches to characterize stem cell‐like gene expression programs and establish prognostically informative signatures [10], validating the transformative potential of multiresolution transcriptomic integration.

The tumor‐associated stroma and immune infiltrate exert deterministic influence on metastatic progression through elaborate intercellular signaling architectures. Accumulating evidence indicates that the microenvironmental landscape critically modulates both metastatic dissemination and therapeutic responsiveness, with single‐cell profiling revealing complex communication networks among cellular constituents [11]. Specific MC subsets establish preferential signaling interactions with stromal and immune partners through coordinated ligand‐receptor engagement, fundamentally reconfiguring the microenvironment to facilitate immunological escape and metastatic spread. Within this intricate signaling landscape, the Eph‐ephrin axis has garnered attention as a key regulator of cellular interactions in malignancy. This receptor family, representing the largest subfamily of receptor tyrosine kinases, mediates bidirectional signaling that governs multiple cellular processes including motility, adhesion, differentiation, and proliferative capacity [12]. Both ephrin ligands and their cognate EPH receptors participate critically in physiological development and pathological processes across diverse malignancies, with EFNA1 implicated in promoting neoplastic expansion through augmentation of cellular proliferation, invasiveness, and metastatic potential [13]. Evidence indicates that ephrin‐A1 functions as a proangiogenic mediator by modulating VEGF‐dependent pathways to facilitate vascular‐dependent metastatic dissemination [14], whereas emerging data suggest that EPH‐ephrin signaling may additionally contribute to immunosuppression by governing tumor‐immune cell interactions [15]. Despite accumulating recognition of EFNA1′s involvement in gastrointestinal carcinogenesis, its precise role in colorectal cancer lymphatic spread, and the cellular mechanisms through which it coordinates microenvironmental remodeling remain insufficiently characterized. Interferon regulatory Factor 9 (IRF9), a transcription factor within the interferon‐stimulated gene Factor 3 complex, has been reported to promote tumorigenesis in several malignancies by modulating IL‐6/STAT3 signaling and extracellular matrix remodeling [16]. However, its role in colorectal cancer metastasis and lymphatic dissemination remains insufficiently characterized.

Despite the progress of both bulk and single‐cell sequencing, a major gap remains: bulk transcriptomics captures clinical phenotypes but lacks cellular resolution, whereas single‐cell data provide cellular heterogeneity but often lack corresponding clinical annotation. Our study addresses this gap by integrating phenotype‐guided bulk information with single‐cell resolution data to pinpoint metastasis‐associated malignant subpopulations. This approach uniquely links clinical outcomes to molecular states within individual cells, thereby revealing mechanistic underpinnings of lymphatic dissemination. In the present investigation, we implemented an integrative multiresolution analytical strategy encompassing tissue‐level transcriptomics, single‐cell gene expression profiling, and computational network modeling to comprehensively dissect the cellular and molecular determinants of lymph node metastasis (LNM) in colorectal cancer. Through integration of phenotype‐guided cell state identification with high‐dimensional coexpression network construction and intercellular communication mapping, we delineated a distinctive MC population driving lymphatic dissemination via IRF9‐orchestrated transcriptional circuitry. Additionally, we identified EFNA1 as a pivotal coordinator of immunosuppressive microenvironmental restructuring within this metastatic population and substantiated its functional relevance through experimental validation and pharmacological sensitivity profiling. These findings establish a comprehensive molecular framework elucidating colorectal cancer LNM and reveal therapeutic susceptibilities within metastasis‐competent MC states, particularly sensitivity to Linifanib‐based interventions.

## 2. Methods

### 2.1. Acquisition and Processing of Bulk Transcriptome Data

We retrieved bulk gene expression profiles together with corresponding clinical metadata from two major public repositories. Primary data originated from the colorectal adenocarcinoma cohort within The Cancer Genome Atlas initiative (TCGA‐CRC; accessible via https://portal.gdc.cancer.gov), supplemented by additional validation datasets sourced from the Gene Expression Omnibus (GEO) database. For high‐throughput RNA sequencing datasets, we established a uniform computational pipeline. Initial raw read counts underwent conversion to transcripts per million (TPM) values, thereby normalizing for variable sequencing depth among samples. Following this, we performed log_2_ transformation on TPM measurements to reduce distributional skewness and achieve variance stabilization throughout the expression spectrum, ultimately enhancing the robustness of subsequent statistical procedures. Microarray‐derived expression measurements necessitated platform‐tailored preprocessing. We executed probe‐to‐gene annotation mapping to convert platform‐dependent probe identifiers into universally recognized gene symbols. When a given gene was interrogated by multiple distinct probes, we computed their mean intensity value to generate a unified expression estimate for that gene. After collapsing to the gene level, we applied cross‐array normalization via the “normalizeBetweenArrays” function from the limma R package, which mitigates systematic technical artifacts arising from different array batches.

### 2.2. Collection and Processing of Single‐Cell Sequencing Data

Single‐cell RNA sequencing datasets were downloaded from the GEO (http://www.ncbi.nlm.nih.gov/geo), encompassing accession identifiers GSE132257, GSE132465, GSE166555, GSE178318, GSE188711, GSE200997, GSE205506, and GSE221575. These resources collectively captured colorectal cancer specimens from diverse independent study cohorts. We utilized the Seurat [17] analytical suite (Version 4.0.4) for comprehensive single‐cell transcriptome interrogation. The workflow commenced by importing raw sequencing outputs through the “Read10X” function, transforming data into a computationally efficient sparse matrix representation (dgCMatrix class) suited for managing high‐dimensional single‐cell datasets. Separate Seurat objects representing individual datasets were unified via the “merge” function, establishing an integrated analytical structure. To circumvent barcode identifier conflicts during dataset combination, we implemented the “RenameCells” function, thereby guaranteeing distinct cell identifiers throughout the consolidated dataset. We enforced stringent quality control measures to eliminate technical artifacts and substandard cellular profiles. The Scrublet algorithm [18] was deployed to algorithmically detect and remove putative doublet events resulting from simultaneous encapsulation of multiple cells. The Scrublet doublet score threshold was set to 0.25. Additional filtering thresholds were established: cells expressing below 100 distinct genes were excluded as likely representing compromised cells or empty droplet captures; conversely, genes detected in under three cells were filtered to minimize noise from sporadic detection. Expression profiles underwent normalization using the “LogNormalize” approach with a scale factor of 10,000, thereby accounting for variations in overall transcript abundance across individual cells. To pinpoint the most biologically informative genes, we applied the “FindVariableFeatures” function to select the 2000 genes demonstrating greatest inter‐cell variability, as these typically encapsulate the most meaningful biological heterogeneity. The “ScaleData” function was subsequently employed to eliminate technical confounding variables, including fluctuations in total unique molecular identifier (UMI) counts and mitochondrial gene expression percentage, with the latter serving as an indicator of cellular stress. This scaling operation centers gene expression measurements and standardizes variance to unity, enabling cross‐gene comparability. Principal component analysis (PCA) was executed on the scaled variable gene expression matrix, retaining the initial 30 principal components to capture dominant variation patterns while maintaining computational tractability. To address batch‐related artifacts stemming from technical disparities across datasets, we implemented the Harmony [19] integration methodology, which projects cells from distinct batches into a unified embedding space while maintaining biological diversity. Harmony [19] batch variable correction was performed using dataset identity as the grouping factor. Postintegration, we employed uniform manifold approximation and projection (UMAP) for two‐dimensional representation of the principal component landscape, enabling intuitive investigation of cellular diversity. Graph‐based unsupervised clustering was performed through consecutive application of the “FindNeighbors” and “FindClusters” functions. The clustering resolution parameter (0.9) was optimized using the “clustree” function, which assesses clustering consistency across varying resolution thresholds. Cell population annotation was achieved by evaluating expression signatures of established lineage‐specific marker genes. Following preliminary broad‐level cell type assignment, epithelial cells were extracted as an independent subset for detailed downstream examination, considering their pivotal involvement in colorectal malignancy biology.

### 2.3. scPAS Analysis for Detecting LNM‐Associated Cell Subsets

We applied the scPAS computational method to identify cell populations exhibiting strong associations with bulk‐derived phenotypic characteristics by integrating bulk transcriptomic information with single‐cell RNA sequencing data. Prior to analysis, raw single‐cell expression matrices underwent quality filtering (cells expressing fewer than 200 genes or showing > 20% mitochondrial gene content were removed) and log‐normalization using the Seurat framework. Highly variable genes were scaled and centered before dimensional reduction. Specimens from patients with LNM and non–lymph node metastasis (nLNM) served as input, encoded as 1 and 0, respectively, for binary classification. The scPAS procedure operated on an imputed expression matrix (KNN‐based imputation) to enhance data density, using the Top 3000 most variable genes for model fitting. Logistic regression with an alpha parameter of 0.03 was used to estimate gene‐specific coefficients, which facilitated phenotype prediction and validation across independent datasets. The model generated a normalized risk score (NRS) for each cell, representing its transcriptional proximity to the LNM phenotype. The NRS served as a quantitative stratification index to guide downstream analyses, including gene coexpression module identification and cell–cell communication network mapping.

### 2.4. High‐Dimensional Weighted Gene Coexpression Network Analysis (hdWGCNA)

We conducted hdWGCNA [20] on single‐cell RNA sequencing data to uncover gene modules linked to LNM. Initially, quality‐filtered single‐cell data were condensed into metacells through averaging expression profiles from small clusters of similar cells, thereby diminishing sparsity and computational demands while maintaining biological variation. The expression matrix then underwent normalization and selection of highly variable genes for network assembly. Scale‐free topology analysis determined an appropriate soft‐thresholding power to approximate a scale‐free network architecture. A signed weighted adjacency matrix was generated by computing Pearson correlation coefficients for all gene pairs and exponentiating them to the selected power. This adjacency matrix was converted into a topological overlap matrix (TOM) to quantify network interconnectedness. Hierarchical clustering performed on TOM‐derived dissimilarity metrics identified gene modules. Dynamic tree cutting was applied to the resulting hierarchical structure to delineate discrete modules, with comparable modules consolidated based on eigengene correlation patterns. Module eigengenes, representing the first principal component of each module′s expression landscape, were calculated to characterize overall module activity across cells. The association between module eigengenes and cellular phenotypes (scPAS+ vs. scPAS− cells) was assessed through correlation analysis and statistical evaluation. Subsequently, genes within modules of interest underwent functional enrichment analysis to ascertain their biological relevance, and module connectivity measures including intramodular connectivity and module membership were computed to pinpoint hub genes orchestrating module dynamics.

### 2.5. Transcription Factor Activity Assessment

We performed single‐cell regulatory network inference and clustering (SCENIC [21]) analysis to characterize transcription factor regulatory architectures and their activities throughout distinct cell populations. The analytical workflow began with quality‐controlled single‐cell expression data, from which coexpression modules were derived using GENIE3 algorithms to establish potential transcription factor‐target gene associations based on expression patterns across cells. These preliminary coexpression modules were refined through RcisTarget to define regulons by analyzing transcription factor binding motifs within regulatory domains of candidate target genes, retaining exclusively those targets displaying significant motif enrichment to ensure bona fide regulatory connections rather than indirect coexpression. For each identified regulon, we calculated the area under the recovery curve (AUC) using the AUCell algorithm to measure regulon activity in single cells by ranking genes according to expression levels and evaluating whether regulon target genes were overrepresented at the ranking apex.

### 2.6. Cell–Cell Communication Network Analysis

We performed intercellular communication analysis utilizing the CellChat [22] algorithm to characterize signaling networks within the tumor microenvironment. Single‐cell RNA sequencing data underwent preprocessing and normalization, with cells stratified by their annotated identities. CellChat leverages a curated repository of ligand‐receptor interactions, encompassing multimeric assemblies and cofactors, to detect potential communication events between cellular populations. For individual ligand‐receptor pairs, the algorithm computed communication probability by integrating ligand expression levels in sender cells with receptor expression in receiver cells, implementing mass action principles to model binding kinetics and employing permutation testing to establish statistical significance. Communication probabilities were consolidated at the pathway level by aggregating probabilities across all ligand‐receptor pairs within identical signaling pathways, thereby identifying predominant pathways mediating intercellular interactions. The comprehensive communication network was characterized by quantifying total interaction numbers and interaction intensity between each cell type pair, with findings presented through circle plots, chord diagrams, and heatmaps to depict the communication architecture. Outgoing and incoming signaling profiles were determined for each cellular population to recognize principal sender and receiver cells within the network. In CellChat, a minimum ligand–receptor expression fraction of 0.1 was used.

### 2.7. Establishment of Stable Cell Lines and Culture Maintenance

The murine colon carcinoma cell line CT26.WT (derived from female BALB/c mouse colon tissue; Cat# TCM37; RRID: CVCL_7254) was obtained from the Cell Bank of Chinese Academy of Sciences (Shanghai, China) on August 20, 2024. Cell line authentication was performed by the supplier using short tandem repeat (STR) profiling prior to shipment, showing 86.7% concordance with the reference profile, which is within the acceptable range for murine cell lines. This cell line is not listed in the International Cell Line Authentication Committee (ICLAC) database of misidentified or contaminated cell lines. Cells were maintained with consistent morphology throughout the experimental period. Mycoplasma contamination was assessed using a PCR‐based detection method in September 2024, confirming negative results. CT26.WT cells were propagated in RPMI‐1640 growth medium supplemented with fetal bovine serum (10% v/v) and antibiotic solution (1% penicillin‐streptomycin) within a humidified incubator maintained at 37°C with 5% CO₂ atmosphere. Stable Efna1‐depleted cell lines were established through lentiviral transduction methodology. Briefly, cells were exposed to lentiviral particles encoding either Efna1‐specific short hairpin RNA or nontargeting scrambled control sequences (designated sh‐NC). Following transduction, antibiotic selection was implemented using puromycin at 2 *μ*g/mL concentration for 7–10 days. Successful knockdown efficiency in resistant clones was subsequently verified through quantitative reverse transcription PCR analysis.

### 2.8. Quantitative Reverse Transcription PCR Analysis

Cellular RNA was isolated employing TRIzol extraction reagent (Beyotime Biotechnology). The concentration and purity of extracted RNA were determined using NanoDrop spectrophotometric measurement. Complementary DNA synthesis was performed from 1 *μ*g total RNA template using the PrimeScript RT Master Mix system (Takara Bio). Real‐time quantitative PCR amplification was conducted on a QuantStudio thermal cycler platform utilizing SYBR Green fluorescent detection chemistry. Expression levels of Efna1 transcript were quantified relative to Gapdh housekeeping gene through the comparative 2^(−*ΔΔ*Ct) method. The oligonucleotide primer sequences employed were as follows: Efna1 sense strand, GGAAGAACAAGGAGTGGAGAC; Efna1 antisense strand, CAGGCAGGGTCAATAATGGG; Gapdh sense strand, GGAGAGTGTTTCCTCGTCCC; Gapdh antisense strand, CCGTTGAATTTGCCGTGAGT.

### 2.9. Cell Viability and Proliferation Quantification

Cellular proliferative capacity was determined using the Cell Counting Kit‐8 colorimetric assay (CCK‐8, Dojindo Molecular Technologies). Experimental cells were seeded into 96‐well microplates at densities ranging from 2000–3000 cells per well in 100 *μ*L complete growth medium. At designated temporal intervals (24, 48, 72, and 96 h postseeding), each well received 10 *μ*L CCK‐8 reagent followed by a 2‐h incubation at 37°C. Optical density measurements were recorded at 450 nm wavelength using a BioTek microplate spectrophotometer. All assays included three technical replicates per condition and were repeated across three independent experimental sessions.

### 2.10. Clonogenic Survival Assay

Long‐term proliferative potential was assessed through colony formation analysis. Cells were seeded at low density (500–1000 cells per well) into 6‐well culture plates and maintained under standard conditions for 10–14 days, with culture medium refreshed at 3‐day intervals. Upon completion of the incubation period, cellular colonies were fixed using 4% paraformaldehyde solution for 15 min, followed by visualization with 0.1% crystal violet staining solution for 20 min. Excess stain was removed through multiple PBS rinses. Colonies exceeding 50 cells were scored under microscopic examination. This experimental protocol was performed in triplicate to ensure reproducibility.

### 2.11. Scratch Wound Closure Assay

Cell migratory behavior was evaluated using an in vitro wound healing model. Cells were seeded into 6‐well plates and allowed to proliferate until achieving 90%–95% confluent monolayers. A standardized linear wound was introduced through the cell layer using a sterile 200 *μ*L pipette tip held perpendicular to the culture surface. Detached cellular debris was removed through two sequential PBS wash steps, and cells were subsequently incubated in serum‐depleted medium. Photographic documentation of wound regions was captured at consistent locations using phase‐contrast inverted microscopy at initial timepoint (0 h) and 24 h postwounding. Wound area measurements were obtained through ImageJ image analysis software, with migration rate calculated using the formula: [(initial area − final area)/initial area] × 100%. Each experimental condition was assessed in triplicate.

### 2.12. Transwell‐Based Migration and Invasion Assays

Cell migratory capacity was quantified using Transwell chamber systems (8 *μ*m pore size, Corning). For standard migration assays, 5 × 10^4^ cells suspended in 200 *μ*L serum‐free medium were transferred to the upper chamber compartment. The lower chamber contained 600 *μ*L complete medium with 10% FBS functioning as a chemoattractant gradient. After 24‐h incubation, nonmigrating cells retained on the upper membrane surface were mechanically removed using cotton‐tipped applicators. Cells that successfully traversed the membrane were fixed with 4% paraformaldehyde and visualized using 0.1% crystal violet staining. Cell enumeration was performed across five randomly selected microscopic fields at 200× magnification. For invasion capability assessment, Transwell membranes were pre‐treated with Matrigel basement membrane matrix (BD Biosciences) diluted 1:8 in serum‐free medium, followed by 4‐h incubation at 37°C to allow gel polymerization. Subsequently, 1 × 10^5^ cells in serum‐free suspension were applied to the upper compartment. The invasion protocol mirrored that of migration assays, except requiring 48‐h incubation before fixation and quantification procedures.

### 2.13. Pharmacological Treatment Regimen

The multikinase inhibitor Linifanib (MedChemExpress, product code HY‐50751) was reconstituted in dimethyl sulfoxide to prepare a 1 mM master stock solution, which was portioned into single‐use aliquots and preserved at −80°C. Fresh working dilutions were prepared in culture medium immediately prior to experimental application, maintaining final DMSO concentration at ≤ 0.1% to avoid solvent toxicity. For combination therapy experiments involving Linifanib and Efna1 depletion, both sh‐NC control and sh‐Efna1 knockdown cells were allowed a 24‐h attachment period following seeding. Subsequently, cells received either 10 *μ*M Linifanib or equivalent volume of vehicle control (0.1% DMSO) and were maintained for experiment‐specific durations: 48 h for CCK‐8 proliferation analysis, 10–14 days for clonogenic assays, 24 h for wound healing studies, and 24–48 h for Transwell migration/invasion experiments. In prolonged incubation protocols, Linifanib‐containing medium was replaced every 2–3 days to maintain stable drug exposure.

### 2.14. Statistical Methodology

All data processing, graphical representation, and statistical computations were performed using *R* statistical computing environment (Version 4.4.0). Relationships between continuous variables were evaluated using Spearman′s rank‐order correlation analysis. Prior to parametric testing, data distributions were assessed for normality. When assumptions of normal distribution and homogeneity of variance were satisfied, intergroup differences were analyzed using Student′s *t*‐test for pairwise comparisons or one‐way analysis of variance for multigroup comparisons. Nonparametric alternatives (Wilcoxon rank‐sum test for two groups, Kruskal–Wallis test for multiple groups) were applied when distributional assumptions were violated. Categorical data were analyzed through chi‐square tests of independence. All experiments incorporated three independent biological replicates to verify consistency of findings. The threshold for statistical significance was established at *p* < 0.05.

## 3. Results

### 3.1. Identification of Cell Subpopulations Associated with Colorectal Cancer LNM Phenotype

We analyzed differential transcriptional perturbations between colorectal cancer patients with and without LNM using bulk transcriptome data. GSEA was performed on differential expression results between bulk samples from LNM and nLNM groups within TCGA‐CRC using log2 fold‐change ranked gene lists as input. LNM samples served as the treatment group and nLNM as control. Results from the Hallmark database (Figure 1a) showed that positively enriched pathways were primarily concentrated in tumor progression‐related processes, including epithelial‐mesenchymal transition, myogenesis, apical junction, and Hedgehog signaling pathway, suggesting that activation of these pathways may promote tumor invasion and metastatic capacity. Additionally, angiogenesis, UV response, and WNT/*β*‐catenin signaling pathways also exhibited positive enrichment. Conversely, negatively enriched pathways mainly involved cell cycle regulation and immune response, such as MYC targets, fatty acid metabolism, inflammatory response, G2/M checkpoint, and interferon response, indicating that these tumor‐suppressive and immune‐related pathways were suppressed during metastasis. KEGG pathway analysis (Figure 1b) further validated these findings. Positively enriched pathways included muscle cell cytoskeletal remodeling, ECM‐receptor interaction, protein digestion and absorption, axon guidance, aldosterone synthesis and secretion, and hormonal signal transduction, all closely related to tumor cell migration, invasion, and microenvironment remodeling. Negatively enriched pathways primarily involved immune functions, such as natural killer cell‐mediated cytotoxicity, autoimmune thyroid disease, influenza A response, amino acid degradation, intestinal immune network, viral protein interaction with cytokines, inflammatory bowel disease, allograft rejection, antigen presentation, and graft‐versus‐host disease, indicating significant immunosuppressive states in LNM patients.

Figure 1(a) Gene set enrichment analysis (GSEA) utilizing the Hallmark gene set collection to evaluate LNM phenotype associations in the TCGA‐CRC cohort. (b) GSEA performed with the KEGG pathway database to examine LNM phenotype characteristics in the TCGA‐CRC cohort. (c) Schematic illustration depicting the integration workflow for single‐cell sequencing datasets. (d) UMAP dimensional reduction visualization displaying eight integrated single‐cell sequencing cohorts. (e) UMAP dimensional reduction plot with annotated cell type identities overlaid on the single‐cell sequencing landscape. (f) Heatmap displaying the expression patterns of canonical marker genes across distinct cell populations.(g) Normalized risk score (NRS) computed by the scPAS algorithm, delineating scPAS− and scPAS+ cell populations. (h) Coefficient values assigned to individual genes by the logistic regression model implemented in the scPAS algorithm. Statistical significance was determined by Student′s *t*‐test (two‐tailed) or one‐way ANOVA as appropriate; *p* < 0.05 was considered significant (*p* < 0.001 where indicated). Data are presented as mean ± SD from three independent biological replicates (*n* = 3). Error bars represent SD.

**Figure:**
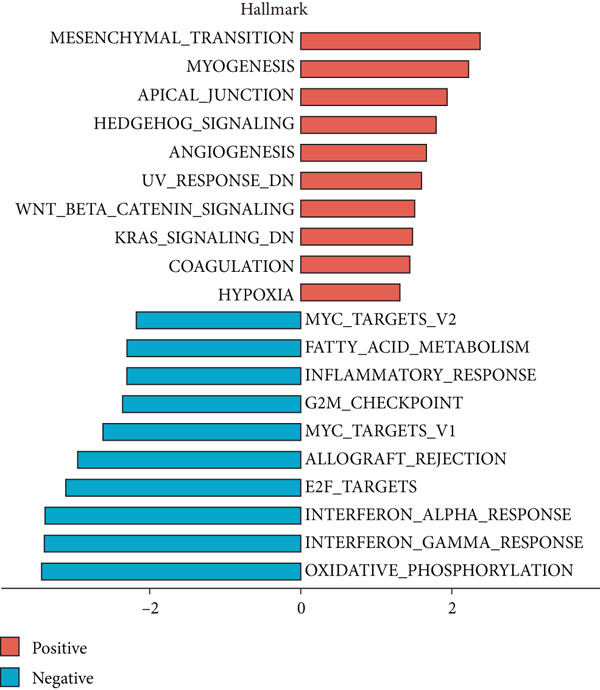
(a)

**Figure:**
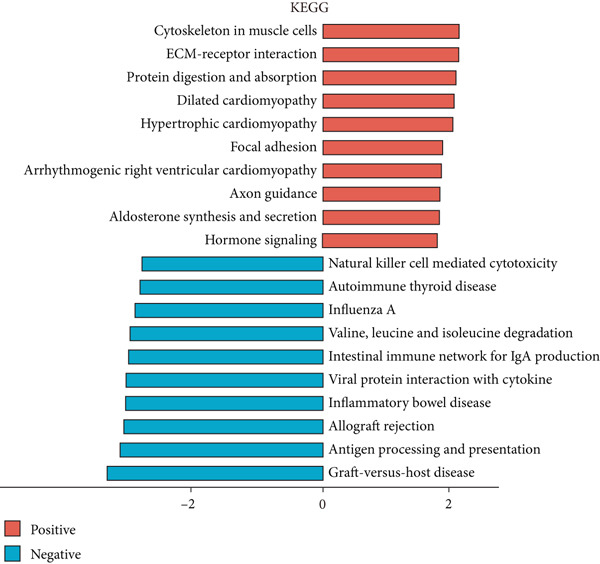
(b)

**Figure:**
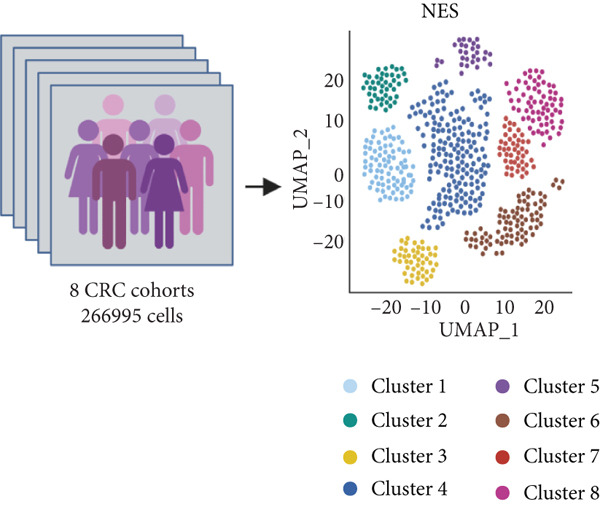
(c)

**Figure:**
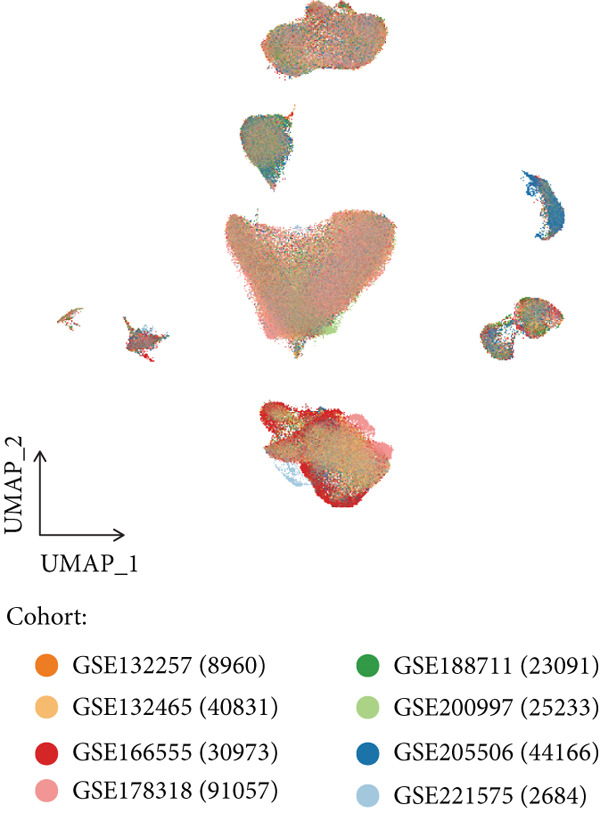
(d)

**Figure:**
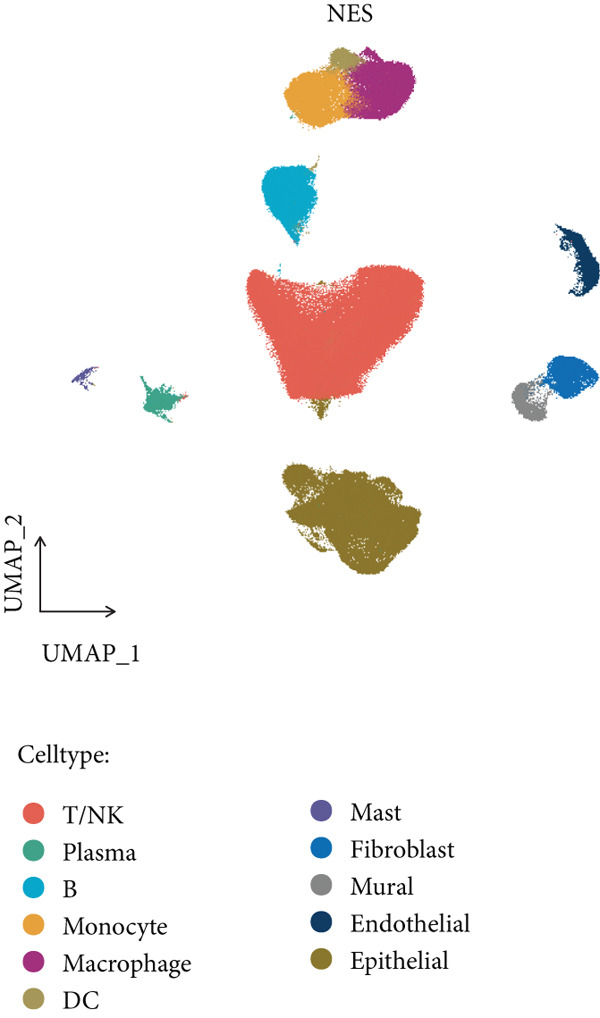
(e)

**Figure:**
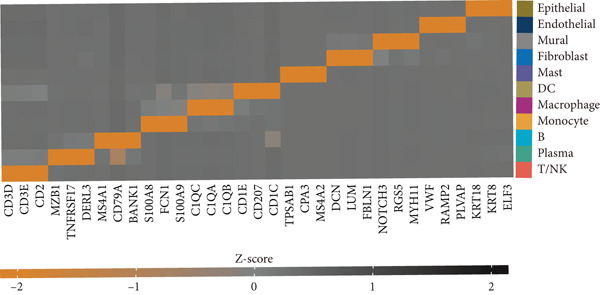
(f)

**Figure:**
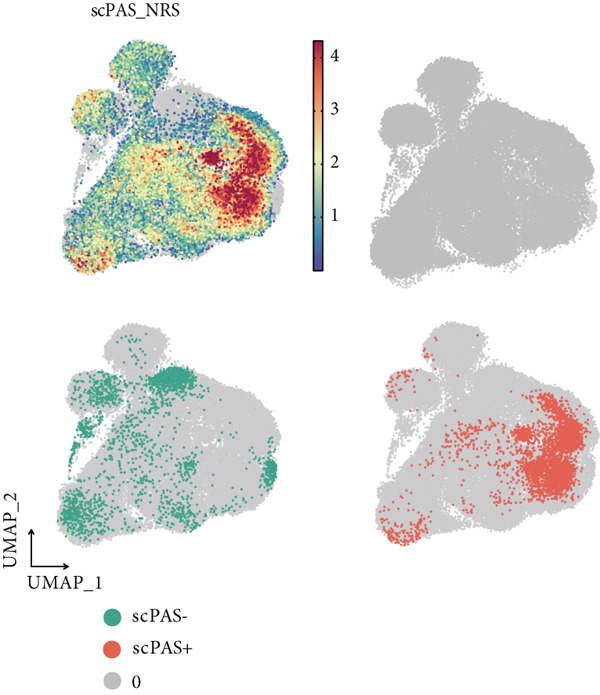
(g)

**Figure:**
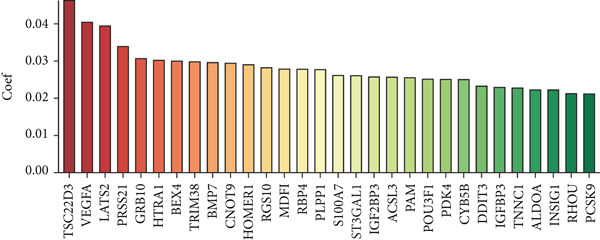
(h)

However, bulk transcriptome analysis remains limited in revealing tumor immune microenvironment alterations. The development of single‐cell sequencing provides opportunities for in‐depth investigation at the individual cell level. To explore potential differences in colorectal cancer LNM patients, we integrated eight colorectal cancer patient tumor tissue single‐cell sequencing cohorts (Figure 1c,d), comprising 266,995 cells that underwent rigorous quality control. All cells were annotated according to classical cell marker genes (Figure 1e,f), including T/NK, plasma, B, monocyte, macrophage, DC, mast, fibroblast, mural, endothelial, and epithelial cells. Most single‐cell sequencing samples had incomplete clinical information. Moreover, due to tumor immune microenvironment complexity and heterogeneity, MCs from LNM phenotype samples do not necessarily all possess metastatic propensity. To identify truly metastatic phenotype‐associated MCs, we employed the scPAS method, which integrates bulk transcriptome and single‐cell transcriptome data to identify cell subpopulations highly correlated with specific phenotypes based on bulk transcriptome data and phenotypic information. Here, we associated LNM and nLNM patients from the TCGA‐CRC cohort as two phenotypes with MCs in single‐cell sequencing data. Using scPAS software, we successfully fitted a logistic regression prediction model that calculated NRS for each cell (Figure 1g). Based on NRS scores, scPAS classified cells into scPAS+ cells highly associated with LNM phenotype and scPAS− cells highly associated with nLNM phenotype. Key genes closely related to colorectal cancer LNM were identified by ranking logistic regression coefficient values (Figure 1h). Results showed that TSC22D3 exhibited the highest coefficient value, suggesting its most important role in promoting LNM. Following closely were genes such as VEGFA, LATS2, and PRSS21, with coefficient values all exceeding 0.030, indicating that high expression of these genes was significantly associated with LNM risk. From a functional perspective, VEGFA as a classical vascular endothelial growth factor demonstrates biological characteristics consistent with tumor metastasis requiring neovascular support; TSC22D3 may participate in metastasis by regulating glucocorticoid response and cell proliferation.

### 3.2. Validation of LNM Prediction Model Stability and Effectiveness

To validate the stability and effectiveness of the NRS scoring system, we included additional transcriptome cohorts. First, we evaluated the association between NRS and LNM status (Figure 2a). Results showed that, in addition to the TCGA‐CRC cohort, NRS scores in LNM patients were significantly higher than in nLNM patients across eight other independent validation cohorts with complete LNM information (GSE71222, GSE41258, GSE39582, GSE39084, GSE29621, GSE18105, GSE28722, and GSE21510). This cross‐cohort consistency fully demonstrates NRS robustness and universal applicability in identifying colorectal cancer LNM patients, unaffected by sample source, detection platform, or population differences. More importantly, we further explored the relationship between NRS scores and patient prognosis (Figure 2b). Through Kaplan–Meier survival curve analysis (Figure 2b), we consistently found across all nine cohorts (TCGA, GSE39582, GSE106584, GSE71187, GSE41258, GSE29621, GSE17536, GSE29621, and GSE28722) that overall survival (OS) of high NRS score patients was significantly shorter than low NRS score patients. Survival curves of high NRS groups showed steeper declining trends, indicating these patients faced higher mortality risk and poorer clinical outcomes. For example, in the TCGA cohort, median survival time of high NRS group patients was markedly shortened, with 5‐year survival rate only approximately 25%, whereas the low NRS group maintained above 50%; similar prognostic differences were reproduced in other validation cohorts. In summary, these cross‐cohort validation results indicate that NRS not only accurately identifies colorectal cancer LNM patients and distinguishes high‐risk and low‐risk populations, but also possesses significant prognostic predictive value, serving as an independent prognostic assessment tool.

Figure 2(a) Box plot comparisons illustrating NRS distribution differences between LNM and nLNM patient samples across multiple independent cohorts. (b) Kaplan–Meier survival curves stratified by NRS across multiple validation cohorts. Statistical significance was determined by Student′s *t*‐test (two‐tailed) or one‐way ANOVA as appropriate; *p* < 0.05 was considered significant (*p* < 0.001 where indicated). Data are presented as mean ± SD from three independent biological replicates (*n* = 3). Error bars represent SD.

**Figure:**
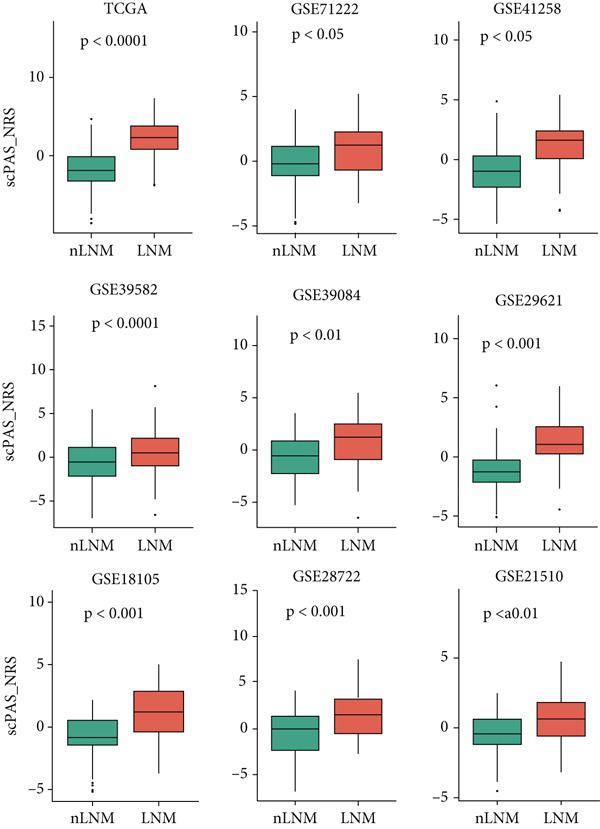
(a)

**Figure:**
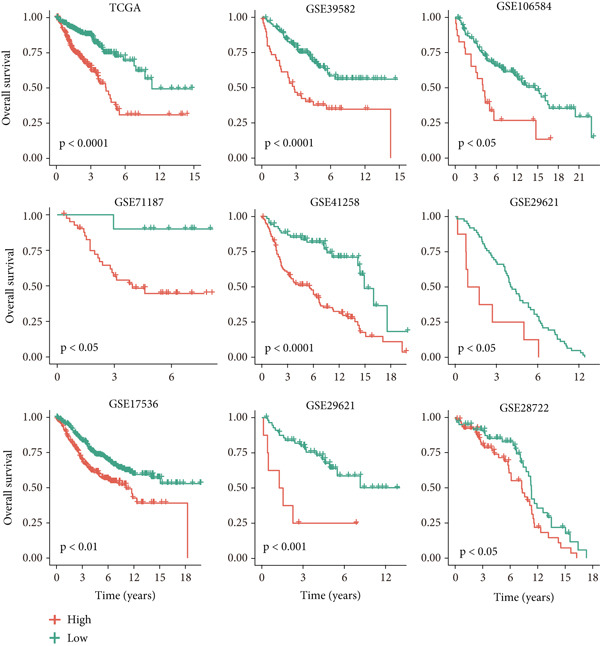
(b)

### 3.3. IRF9 May Act as a Potential Upstream Regulator Driving MC‐M2 Module Activation and Shaping the Transcriptional Program of scPAS+ cells

Cell phenotype formation is driven by coexpressed gene modules. To identify gene modules highly associated with scPAS+ cells, we performed hdWGCNA on single‐cell sequencing data. Through systematic network construction and module identification, we identified 15 high‐quality gene coexpression modules (MC‐M1 to MC‐M15), which displayed a clear hierarchical structure in dendrogram clustering (Figure 3a). Each module was identified by different colors, representing coordinated expression networks of functionally related genes. Through UMAP dimensionality reduction visualization analysis (Figure 3b), we found that the MC‐M2 module (purple) exhibited significant activation in scPAS+ cells, with high expression regions highly overlapping with the spatial distribution of scPAS+ cells. Further analysis revealed that the MC‐M2 module primarily includes ANXA1, S100A11, CSTB, S100A4, S100A2, KLK10, CAV2, LGALS1, and others (Figure 3c). Notably, this module contains multiple S100 family proteins (S100A11, S100A4, S100A2), suggesting the MC‐M2 module plays important roles in stress response, survival, and migratory capacity of scPAS+ cells (Figure 3d). To more precisely quantify the association strength between different gene modules and scPAS+ cells, we used the Top 100 differentially expressed signature genes of scPAS+ cells as a gene set to perform enrichment scoring on each coexpression module. Subsequently, we quantified the correlation between this scPAS+ signature score and the 15 gene modules (Figure 3e). Results showed that the MC‐M2 module exhibited the highest positive correlation with scPAS+ cell signature scores, whereas MC‐M11, MC‐M6, MC‐M8, and other modules showed negative or weak correlations. This finding further confirms that the MC‐M2 module is the core gene module of the scPAS+ cell phenotype, with its activation state directly determining the molecular characteristics and functional state of scPAS+ cells. Gene module formation and activation are often dominated by powerful transcription factors (TFs). Therefore, we further employed SCENIC analysis to systematically quantify key transcription factor activities. We identified multiple TFs with high activity in scPAS+ cells. Importantly, we found that IRF9 activity exhibited the highest positive correlation with MC‐M2 module expression levels (Figure 3f). IRF9 acts as a master regulator driving MC‐M2 module activation, thereby shaping the molecular phenotype of scPAS+ cells. To validate the role of IRF9 in scPAS+ cell formation and LNM, we further analyzed the relationship between IRF9 expression levels and LNM status and patient prognosis in multiple bulk transcriptome cohorts. As shown in Figure 3g, in two independent cohorts (GSE41258 and GSE39582), IRF9 activity in LNM patients was significantly higher than in nLNM patients, consistent with single‐cell level observations. Additionally, survival analysis showed (Figure 3h) that across multiple cohorts including TCGA, GSE17536, and GSE17537, OS of high IRF9 expression patients was significantly shorter than low IRF9 expression patients, indicating that IRF9 not only participates in LNM processes but is also closely associated with poor prognosis in colorectal cancer patients. In summary, by integrating hdWGCNA, SCENIC transcription factor activity analysis, and multicohort validation, we systematically revealed the molecular mechanism by which IRF9 drives scPAS+ cell phenotype formation through activation of the MC‐M2 coexpression module.

Figure 3(a) Dendrogram displaying hierarchical clustering of gene modules identified through hdWGCNA. (b) UMAP visualization showing the expression scores of individual gene modules across the cellular landscape. (c) UMAP plots annotated with gene expression patterns for selected gene modules. (d) Bar graphs demonstrating the expression preference of each module across cells with distinct phenotypic characteristics. (e) Correlation heatmap depicting associations between individual gene modules and scPAS+ cell features. (f) Scatter plots showing correlations between various transcription factor activities and M2 module expression levels. (g) Comparative analysis of IRF9 activity between LNM and nLNM patient samples. (h) Kaplan–Meier survival analysis stratified by IRF9 activity across multiple cohorts. Statistical significance was determined by Student′s *t*‐test (two‐tailed) or one‐way ANOVA as appropriate; *p* < 0.05 was considered significant (*p* < 0.001 where indicated). Data are presented as mean ± SD from three independent biological replicates (*n* = 3). Error bars represent SD.

**Figure:**
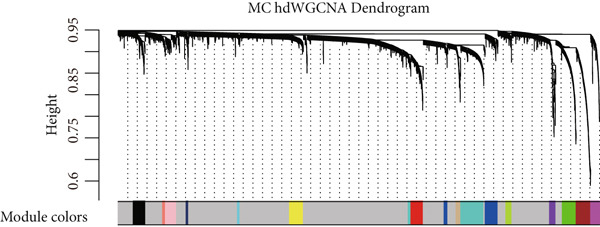
(a)

**Figure:**
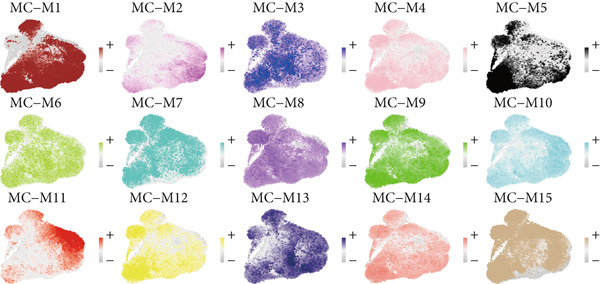
(b)

**Figure:**
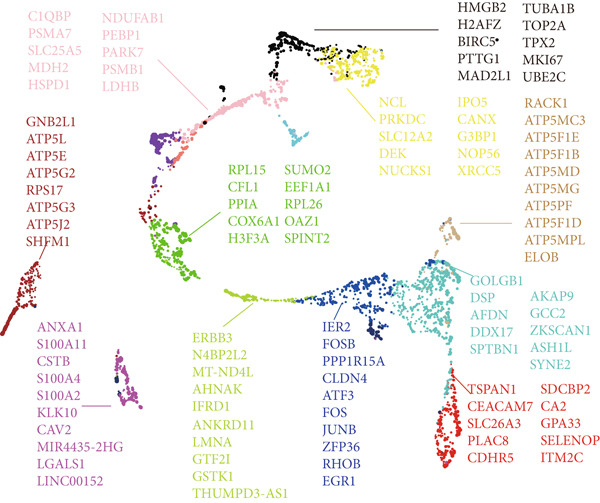
(c)

**Figure:**
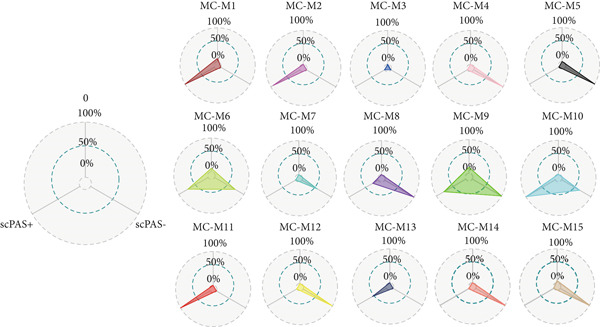
(d)

**Figure:**
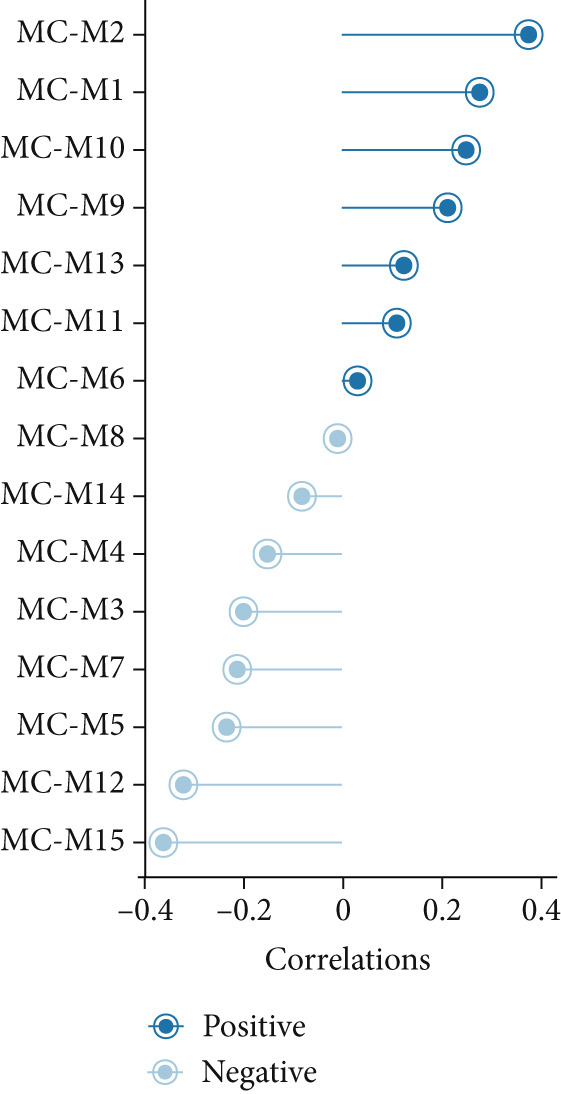
(e)

**Figure:**
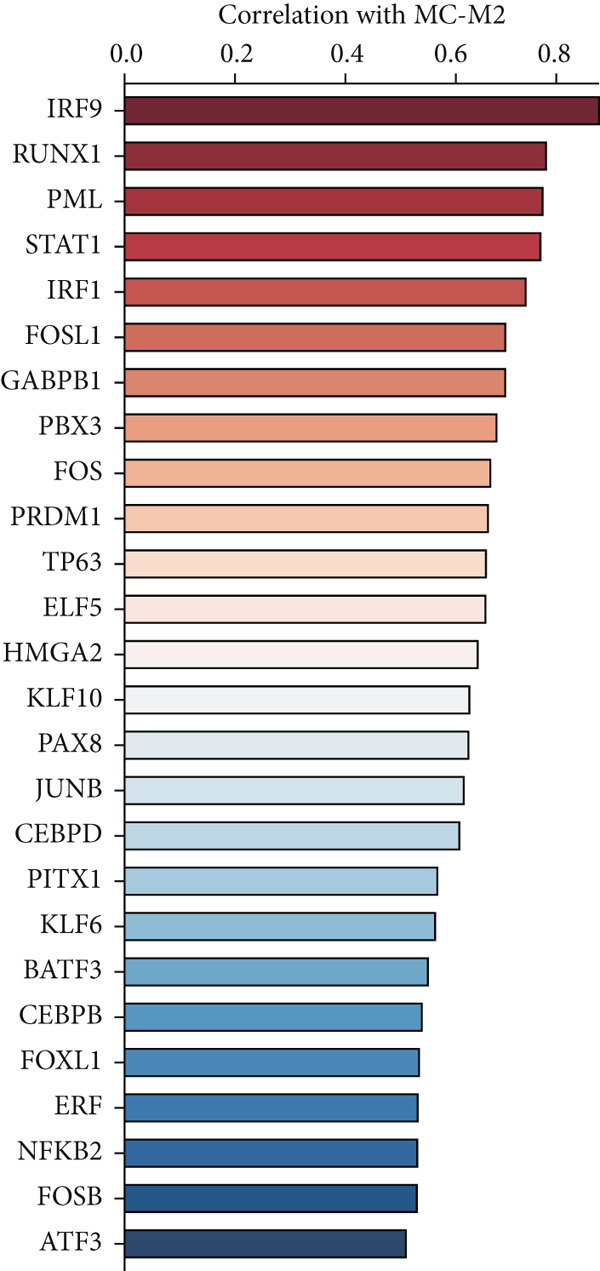
(f)

**Figure:**
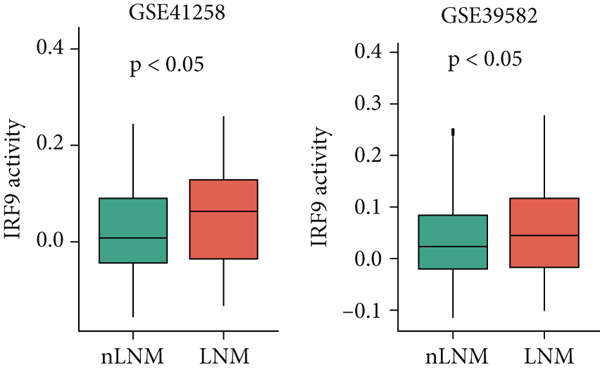
(g)

**Figure:**
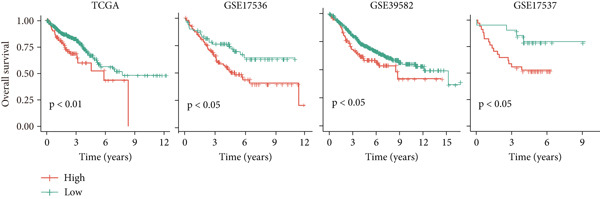
(h)

### 3.4. scPAS+ Cells Construct Immunosuppressive Microenvironment by Remodeling Cell Communication Networks

To deeply understand the role of scPAS+ MC in the tumor microenvironment, we systematically analyzed ligand‐receptor–mediated cell communication networks among different cell subpopulations. The cell–cell communication network diagram (Figure 4a) displays complex interaction relationships among various cell types in the tumor microenvironment, where scPAS+ cells as a MC subpopulation establish extensive and dense communication connections with immune cells and stromal cells, suggesting their central role in microenvironment remodeling. Through quantitative analysis of cell–cell interaction numbers (Figure 4b), we found that communication activity between scPAS+ MCs and multiple cell types was significantly higher than scPAS− cells. Specifically, scPAS+ cells showed the highest number of interactions with fibroblasts. In communications with endothelial cells, scPAS+ cells showed 79 interactions versus 72 for scPAS− cells. In interactions with mural cells, scPAS+ cells showed 73 interactions versus 65 for scPAS− cells. Additionally, communication numbers between scPAS+ cells and macrophages, monocytes, and DCs were also enhanced. This comprehensive communication enhancement indicates that scPAS+ MCs actively establish dense communication networks to recruit and educate stromal and immune cells, systematically remodeling the tumor microenvironment to support invasion, metastasis, and immune evasion. Through ligand‐receptor pair analysis (Figure 4c), we identified key signaling pathways significantly enriched in communications between scPAS+ cells and other cells. Results showed that scPAS+ MC highly express multiple proangiogenic factors, including VEGFA‐VEGFR2/VEGFR1 ligand‐receptor pairs, which are significantly activated in communications between scPAS+ cells and endothelial cells. In contrast, VEGF signaling activity in scPAS− cells was markedly weaker. This suggests that scPAS+ MC drive tumor neovascularization and lymphangiogenesis through continuous secretion of VEGF family factors, establishing structural channels for tumor cell nutrient supply and LNM. Figure 4d further demonstrates high enrichment of VEGFA‐KDRC signaling in scPAS+ cells, validating differential activation of this proangiogenic signal. Notably, communications between scPAS+ MC and fibroblasts, endothelial cells, and mural cells were highly enriched in integrin signaling pathways, including LAMB3‐ITGB1, LAMB3‐ITGAV, LAMC2‐ITGA7/ITGB1, LAMA3‐ITGA6/ITGB1/ITGB4, and other ligand‐receptor combinations (Figure 4c). These integrin signals exhibited significant activation states in scPAS+ cells, whereas activity was markedly attenuated in scPAS− cells. Figure 4d trajectory visualization further confirmed enrichment of integrin signal pairs such as LAMB3‐ITGB1, LAMB3‐ITGAV, LAMC2‐CD44, and LAMA3‐ITGAV in scPAS+ cells. Integrin‐mediated cell‐extracellular matrix adhesion and signal transduction are key mechanisms of tumor cell invasion and migration. By upregulating integrin and ligand expression, scPAS+ MC enhance physical connections and bidirectional signal exchange with stromal cells, thereby gaining stronger matrix remodeling capacity, invasiveness, and metastatic potential. This enhanced cell–matrix interaction enables scPAS+ cells to breach tissue barriers and disseminate to distant sites such as lymph nodes.

Figure 4(a) Global intercellular communication network landscape generated by the CellChat algorithm. (b) Quantification of communication interaction numbers between different cell type pairs. (c) Dot plot representation displaying cell–cell communication strength across cell populations. (d) Chord diagram illustrating dominant intercellular signaling pathways. (e) Kaplan–Meier survival analysis of key ligand expression in the TCGA‐CRC cohort. Statistical significance was determined by Student′s *t*‐test (two‐tailed) or one‐way ANOVA as appropriate; *p* < 0.05 was considered significant (*p* < 0.001 where indicated). Data are presented as mean ± SD from three independent biological replicates (*n* = 3). Error bars represent SD.

**Figure:**
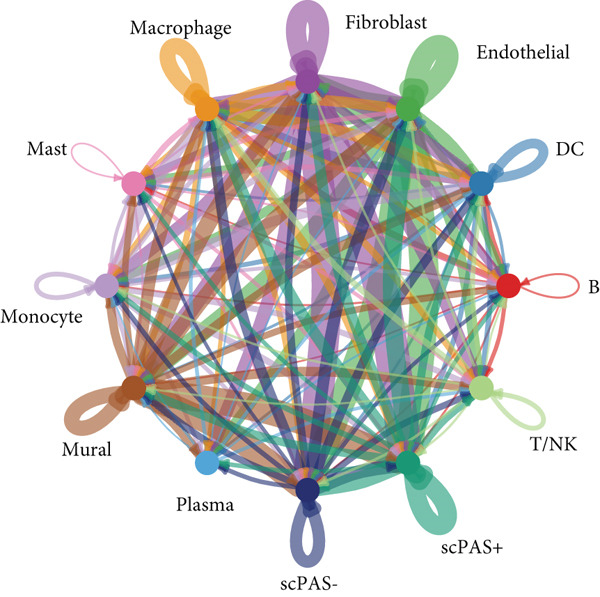
(a)

**Figure:**
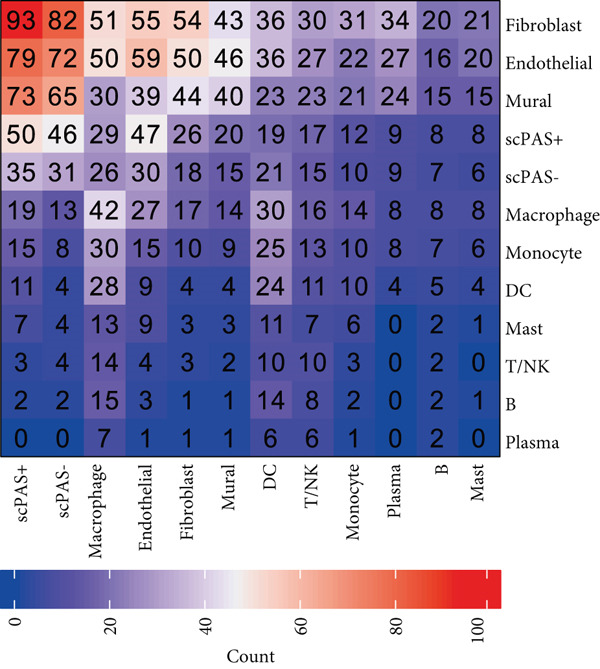
(b)

**Figure:**
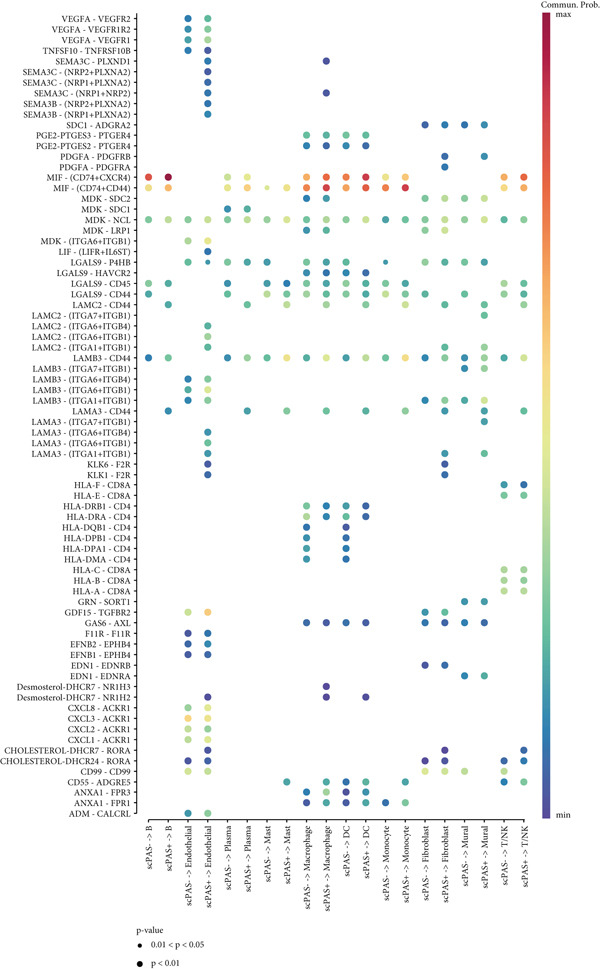
(c)

**Figure:**
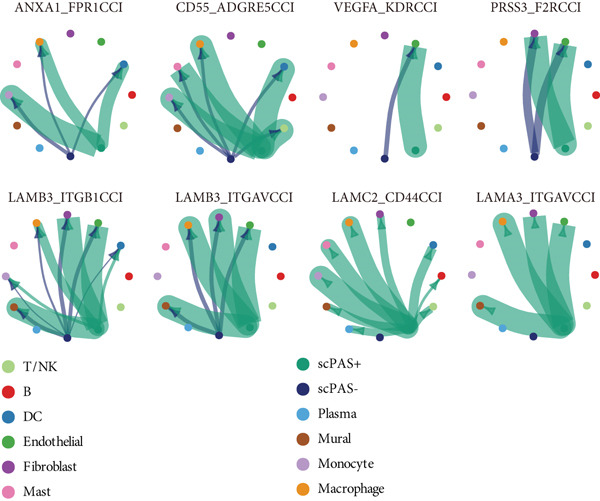
(d)

**Figure:**
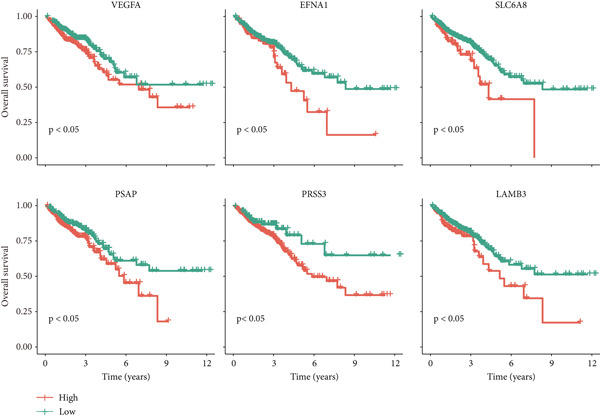
(e)

To validate the clinical significance of key molecules in the scPAS+ cell communication network, we analyzed the relationship between these molecules and patient prognosis in multiple independent cohorts (Figure 4e). Survival analysis showed that patients with high expression of VEGFA, EFNA1, SLC6A8, PSAP, PRSS3, and LAMB3 had significantly shorter OS than low expression patients. These molecules participate in angiogenesis (VEGFA and EFNA1), metabolic reprogramming (SLC6A8), lipid metabolism and immune regulation (PSAP), proteolysis (PRSS3), and cell–matrix adhesion (LAMB3), respectively. Their high expression association with poor prognosis not only validates the clinical relevance of the scPAS+ MC communication network but also suggests these communication axes as potential therapeutic targets. Blocking interactions between scPAS+ cells and the microenvironment may effectively inhibit LNM and improve patient prognosis. In summary, scPAS+ MC, as an immunosuppression‐related MC subpopulation, systematically remodel the tumor microenvironment by constructing far denser and complex intercellular communication networks than scPAS− cells.

### 3.5. Comprehensive Association Analysis of EFNA1 Expression With Colorectal Cancer Clinicopathological Features, Immune Microenvironment, and Genomic Characteristics

Among numerous active communication molecules, we noted that EFNA1, as a key ligand of the Ephrin family, is not only significantly enriched in communications between scPAS+ cells and other cell types, but more importantly, high EFNA1 expression is closely associated with poor prognosis in colorectal cancer patients. The Ephrin‐Eph signaling pathway plays important regulatory roles in embryonic development, angiogenesis, neural guidance, and tumor progression. As a key ligand of this pathway, high expression of EFNA1 in scPAS+ MC suggests it may play central roles in mediating cell–cell interactions, promoting vascular and lymphatic vessel formation, and remodeling the immune microenvironment. Given EFNA1′s prominent position in the scPAS+ cell communication network and its significant association with patient prognosis, we selected EFNA1 as a representative molecule for in‐depth multidimensional analysis to comprehensively evaluate its clinical significance and molecular mechanisms in colorectal cancer development. To comprehensively evaluate the clinical significance and molecular characteristics of EFNA1 in colorectal cancer, we systematically analyzed correlations between EFNA1 expression levels and multiple clinical indicators, immune‐related gene expression, and genomic mutation features. First, we validated the relationship between EFNA1 expression and tissue type and metastatic status in multiple independent cohorts (Figure 5a). Results showed that in the TCGA‐CRC cohort, EFNA1 expression levels in tumor tissues were significantly higher than in normal tissues, a finding consistently validated in GSE87211 and GSE41258 validation cohorts. More importantly, when comparing different metastatic states, we found that in the TCGA‐CRC cohort, M1 stage (distant metastasis) patients exhibited significantly higher EFNA1 expression than M0 stage (no distant metastasis) patients, a trend similarly confirmed in GSE64256 and GSE71222 validation cohorts. These results indicate that EFNA1 is not only upregulated during tumorigenesis but further elevated during tumor progression to distant metastasis, suggesting it may be a key molecule driving colorectal cancer invasion and metastasis. To deeply understand the relationship between EFNA1 and the tumor immune microenvironment, we divided patients into four groups (Q1: highest expression to Q4: lowest expression) based on EFNA1 expression quartiles and analyzed expression patterns of different immune‐related genes in each group (Figure 5b). Heatmap results revealed highly complex immune gene expression patterns, encompassing costimulatory molecules, immune checkpoint ligands and receptors, chemokines, cytokines, antigen presentation molecules, and other categories. Regarding costimulatory molecules, the EFNA1 low expression group (Q3–Q4) showed relatively high expression of multiple costimulatory molecules such as CD80, CD86, and ICOSLG, whereas these molecules were relatively low in the EFNA1 high expression group (Q1–Q2), suggesting high EFNA1 expression may be associated with attenuated costimulatory signals. More critically, immunosuppressive ligands and receptors showed distinct expression patterns. Unexpectedly, multiple classical immune checkpoint molecules such as PDCD1LG2, CTLA4, LAG3, and TIGIT showed relatively higher expression in the EFNA1 low expression group (Q3–Q4), whereas relatively lower in the EFNA1 high expression group (Q1–Q2). This unexpected finding suggests EFNA1 may mediate immunosuppression through mechanisms distinct from classical immune checkpoints, or that high EFNA1 expression defines a colorectal cancer subtype with relatively low immune checkpoint expression that achieves immune evasion through alternative pathways. Regarding chemokines and cytokines, expression patterns showed significant heterogeneity. Pro‐inflammatory chemokines such as CXCL9, CXCL10, and CXCL11 showed relatively higher expression in the EFNA1 low expression group but lower in the EFNA1 high expression group, consistent with immune checkpoint expression patterns, suggesting EFNA1 low expression tumors may have stronger immune infiltration and inflammatory responses. Other chemokines such as CCL2, CXCL8, and CXCL12 exhibited complex variation patterns across different EFNA1 expression groups. Remarkably, across all five independent cohorts, EFNA1 expression showed significant negative correlation with CD274 expression (Figure 5c). This consistent cross‐cohort negative correlation indicates that tumors with high EFNA1 expression actually have relatively low PD‐L1 expression levels. This finding aligns with the pattern observed in Figure 5d where immune checkpoints were relatively low in the EFNA1 high‐expression group, suggesting that EFNA1 high‐expression tumors may represent an immune evasion subtype independent of the classical PD‐1/PD‐L1 axis. These tumors may construct immunosuppressive microenvironments through other mechanisms such as angiogenesis, matrix remodeling, metabolic reprogramming, or recruitment of immunosuppressive cells (e.g., MDSCs and M2 macrophages) rather than primarily relying on immune checkpoint upregulation. Additionally, we found decreased abundance of infiltrating T cells in patient samples with high EFNA1 expression. Although EFNA1 high tumors display low PD‐L1 expression, this does not indicate immune activation; instead, they represent an “immune‐cold” subtype characterized by low infiltration and checkpoint expression, consistent with alternative immune‐evasion pathways.

Figure 5(a) Comparative analysis of EFNA1 expression across normal tissue, tumor tissue, and M0 versus M1 stage samples. (b) Heatmap depicting the expression landscape of immune‐related molecules, along with methylation status, amplification, and deletion events, stratified by EFNA1 expression quartiles (Q1: highest expression to Q4: lowest expression). (c) Scatter plot correlation analysis between EFNA1 and CD274 expression levels across multiple independent cohorts. (d) Comparative analysis of infiltrating T cell populations between EFNA1‐high and EFNA1‐low expression patient groups. (e) Association analysis between EFNA1 expression levels and MSI status. (f) Correlation analysis examining the relationship between EFNA1 expression and genomic instability signatures. Statistical significance was determined by Student′s *t*‐test (two‐tailed) or one‐way ANOVA as appropriate; *p* < 0.05 was considered significant (*p* < 0.001 where indicated). Data are presented as mean ± SD from three independent biological replicates (*n* = 3). Error bars represent SD.

**Figure:**
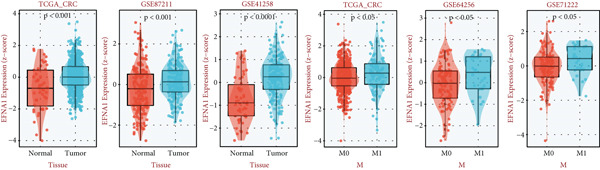
(a)

**Figure:**
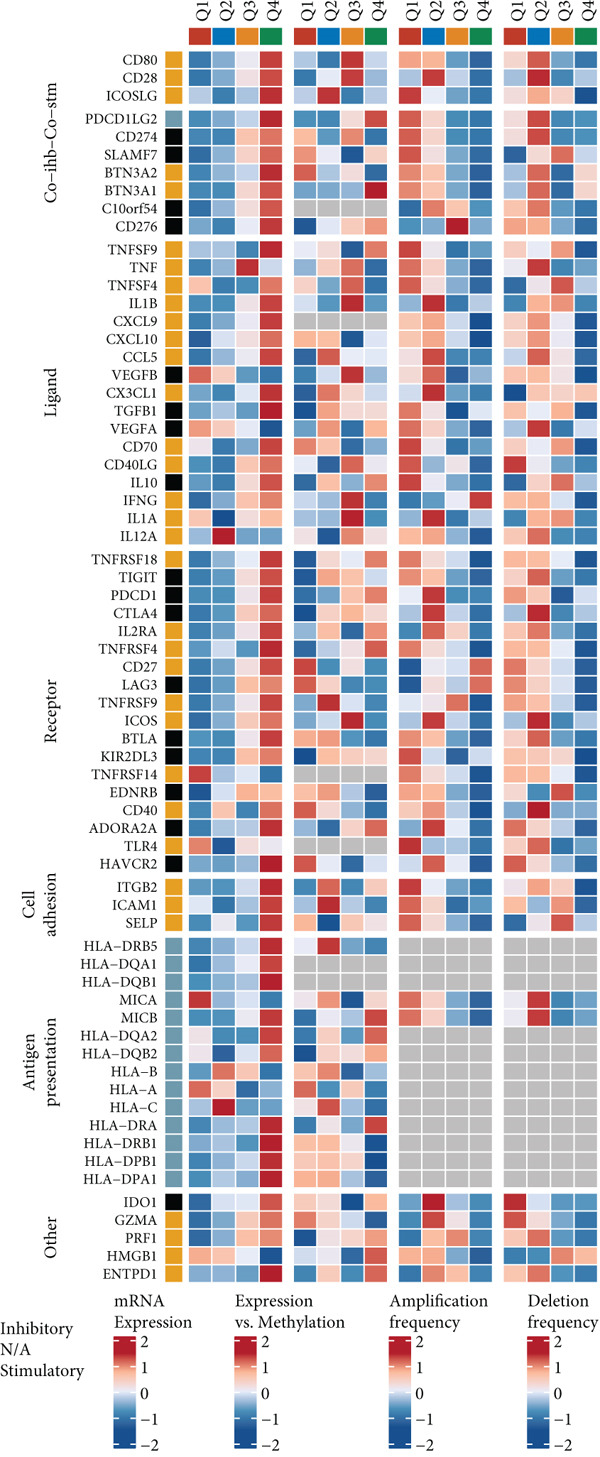
(b)

**Figure:**
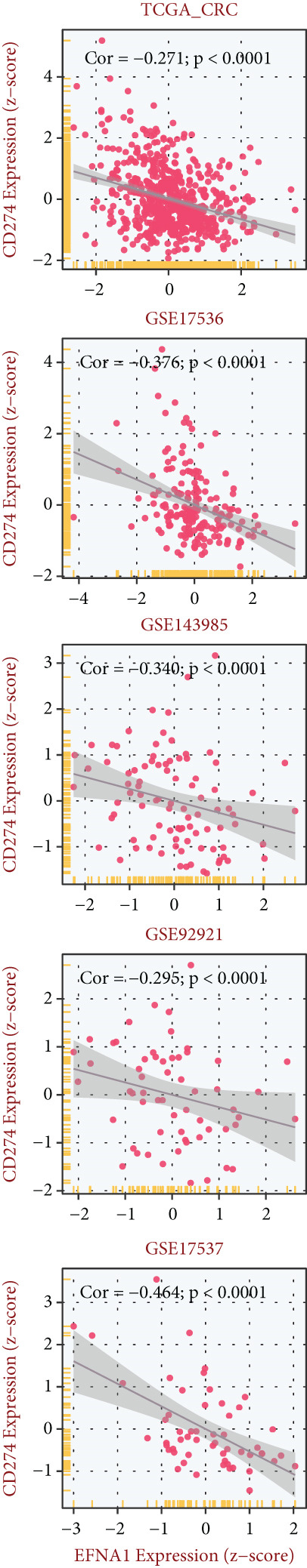
(c)

**Figure:**
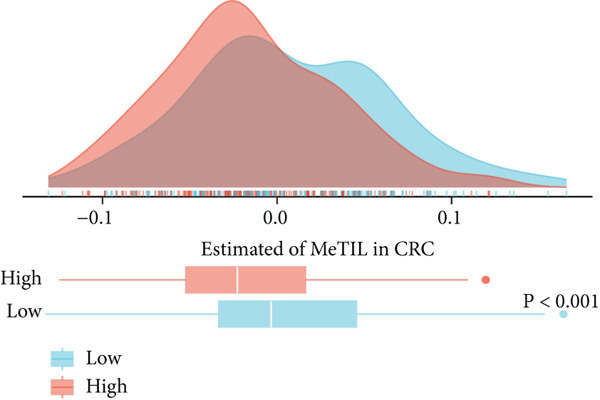
(d)

**Figure:**
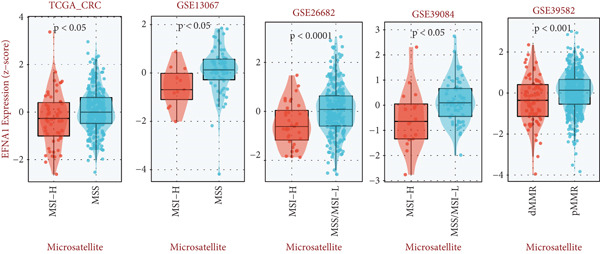
(e)

**Figure:**
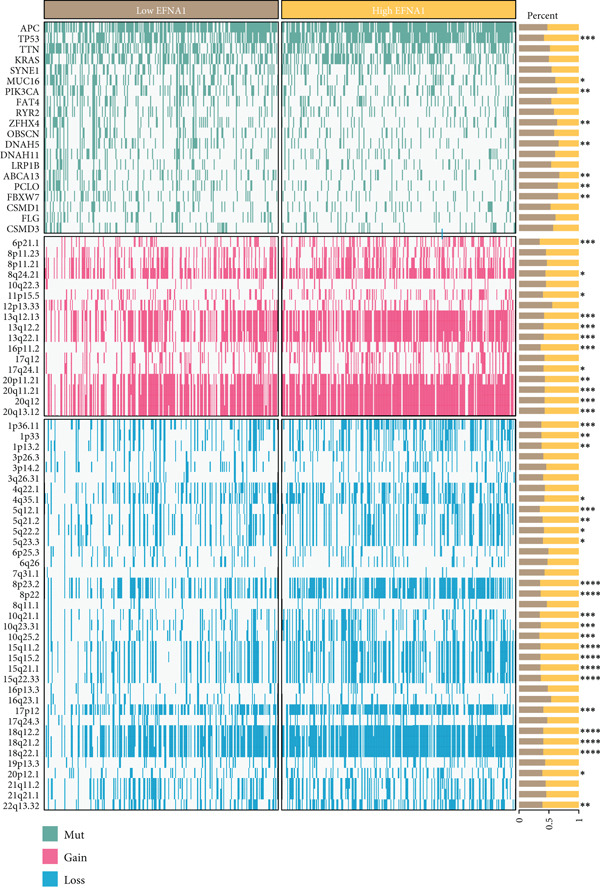
(f)

Microsatellite instability (MSI) is an important molecular subtype marker of colorectal cancer, closely related to immunotherapy response. We analyzed the relationship between EFNA1 expression and MSI status in multiple cohorts (Figure 5e). Results showed that in the TCGA‐CRC cohort, EFNA1 expression levels in microsatellite stable (MSS) tumors were significantly higher than in microsatellite unstable (MSI) tumors (*p* < 0.05), a trend consistently validated in GSE13067, GSE39582, GSE26682, GSE39084, and other validation cohorts. MSI tumors typically have high tumor mutational burden (TMB) and strong immune infiltration, respond well to immune checkpoint inhibitor therapy, and often express higher levels of PD‐L1 and other immune checkpoint molecules. High EFNA1 expression in MSS tumors and negative correlation with PD‐L1 further supports the view that EFNA1 high expression tumors represent an immunologically “cold” subtype. This subtype is characterized by low immune checkpoint expression and low immune infiltration but achieves immune evasion and promotes metastasis through EFNA1‐mediated alternative mechanisms.

Furthermore, we systematically analyzed associations between EFNA1 expression levels and common gene mutations in colorectal cancer (Figure 5f). The waterfall plot displayed mutation, amplification, and deletion patterns of various genes in patient cohorts arranged by EFNA1 expression from low to high. Results showed that some genes such as FBXW7, SMAD4, PIK3CA, and BRAF exhibited higher mutation frequencies in the EFNA1 low expression group, whereas mutation patterns of certain genes such as APC and TP53 were more concentrated in the EFNA1 high expression group. Beyond point mutations, gene amplifications and deletions also showed differential distributions in patients with different EFNA1 expression levels, with amplification events of certain genes more common in the EFNA1 high‐expression group, whereas deletions of some genes were enriched in the low‐expression group.

### 3.6. Efna1 Knockdown Suppresses Colorectal Cancer Cell Malignant Phenotypes and Enhances Linifanib Drug Sensitivity

To validate the functional role of EFNA1 in colorectal cancer progression, we constructed stable Efna1 knockdown colorectal cancer cell lines in the CT26 colon cancer cell line and performed systematic in vitro functional experiments. First, we verified Efna1 knockdown efficiency by qRT‐PCR (Figure 6a). Results showed that compared with the negative control group (sh‐NC), Efna1 mRNA expression levels in the Efna1 knockdown group (sh‐Efna1) significantly decreased, confirming successful stable knockdown cell line construction. Subsequently, we evaluated the effect of Efna1 knockdown on cell proliferation capacity through CCK‐8 assay (Figure 6b). Results showed that proliferation rates of sh‐Efna1 group cells were significantly lower than sh‐NC group at all time points, indicating Efna1 knockdown significantly inhibited colorectal cancer cell proliferation. Colony formation assay results showed that the sh‐Efna1 group formed significantly fewer colonies than the sh‐NC group (Figure 6c), confirming that Efna1 knockdown not only inhibits short‐term proliferation but also significantly reduces cell clonogenic and long‐term survival capacity. Wound healing assay showed that after 24 h, wound healing was markedly faster in the sh‐NC group than the sh‐Efna1 group, indicating Efna1 knockdown significantly weakened cell migration ability (Figures 6d,e). Transwell migration assay showed (Figure 6f) that the number of cells passing through the Transwell membrane was significantly fewer in the sh‐Efna1 group than the sh‐NC group. Invasion assay results (Figure 6f) showed that the number of cells passing through Matrigel was similarly significantly reduced in the sh‐Efna1 group. These results indicate that Efna1 not only promotes basal cell migration ability but more importantly enhances cellular capacity to degrade extracellular matrix and traverse tissue barriers, highly consistent with biological characteristics of MCs needing to breach basement membranes and lymphatic vessel walls during LNM. To explore the relationship between Efna1 expression and drug sensitivity, we analyzed correlations between Efna1 expression and various antitumor drug responses in the GDSC drug screening database (Figure 6g). The heatmap displayed correlations between Efna1 expression levels and sensitivity to dozens of drugs. Notably, among numerous drugs, we found that high Efna1 expression positively correlated with resistance to multiple targeted drugs, particularly Linifanib. We performed CCK‐8 assays with combined Linifanib treatment in sh‐NC and sh‐Efna1 cells (Figure 6h). Results showed that with Linifanib treatment alone, sh‐NC group cells exhibited certain growth inhibition, but sh‐Efna1 group cells demonstrated significantly enhanced sensitivity to Linifanib. Colony formation assay (Figure 6i) showed that colony formation numbers in the sh − Efna1 + Linifanib group were far lower than the sh‐NC group treated with Linifanib alone. Wound healing assay further demonstrated that under Linifanib treatment conditions, wound healing rates in the sh − Efna1 + Linifanib group were significantly lower than the sh − NC + Linifanib group. Transwell migration and invasion assays under Linifanib treatment conditions similarly showed synergistic effects (Figures 6k,l). Migrating cell numbers (Figure 6k) and invading cell numbers (Figure 6l) in the sh − +Linifanib group were both significantly lower than the sh − NC + Linifanib group. In summary, in vitro functional experiments systematically validated the critical role of Efna1 in maintaining colorectal cancer malignant phenotypes. Efna1 knockdown suppresses colorectal cancer cell malignant phenotypes and enhances Linifanib drug sensitivity.

Figure 6(a) Quantitative PCR validation of Efna1 mRNA expression levels comparing sh‐Efna1 knockdown cells to sh‐NC control cells. (b) Growth curve analysis showing absorbance at 450 nm over time from CCK‐8 proliferation assays in sh‐Efna1 versus sh‐NC cells. (c) Representative images and quantification of colony formation capacity in sh‐Efna1 knockdown compared with sh‐NC control cells. (d,e) Representative microscopy images and quantitative analysis of wound closure percentage at 24 h postscratch in sh‐Efna1 versus sh‐NC cells. (f) Representative images and quantification of Transwell migration and invasion assays comparing sh‐Efna1 and sh‐NC cells. (g) Correlation analysis between Efna1 expression levels and drug sensitivity profiles from the GDSC pharmacogenomic database. (h) CCK‐8 proliferation assay measuring absorbance at 450 nm in cells treated with combined sh‐Efna1 knockdown plus Linifanib versus sh‐NC plus Linifanib. (i) Representative images and quantification of colony formation in combined treatment groups (sh − Efna1 + Linifanib vs. sh − NC + Linifanib). (j) Wound healing assay quantification at 24 h comparing combined treatment groups (sh − Efna1 + Linifanib vs. sh − NC + Linifanib). (k,l) Representative images and quantification of migrated and invaded cells in Transwell assays under combined treatment conditions (sh − Efna1 + Linifanib vs. sh − NC + Linifanib). Statistical significance was determined by Student′s *t*‐test (two‐tailed) or one‐way ANOVA as appropriate; *p* < 0.05 was considered significant (*p* < 0.001 where indicated). Data are presented as mean ± SD from three independent biological replicates (*n* = 3). Error bars represent SD.

**Figure:**
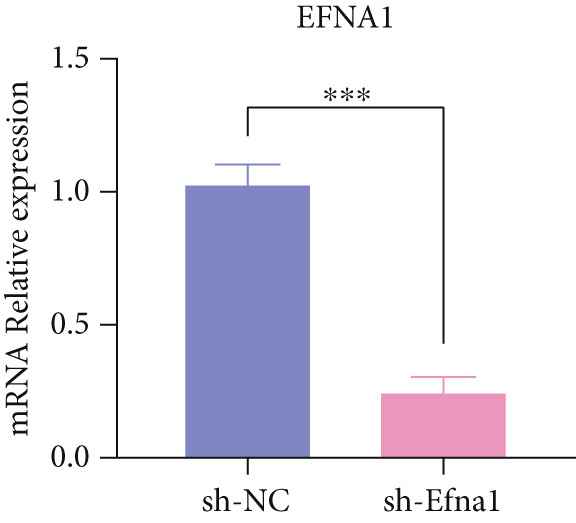
(a)

**Figure:**
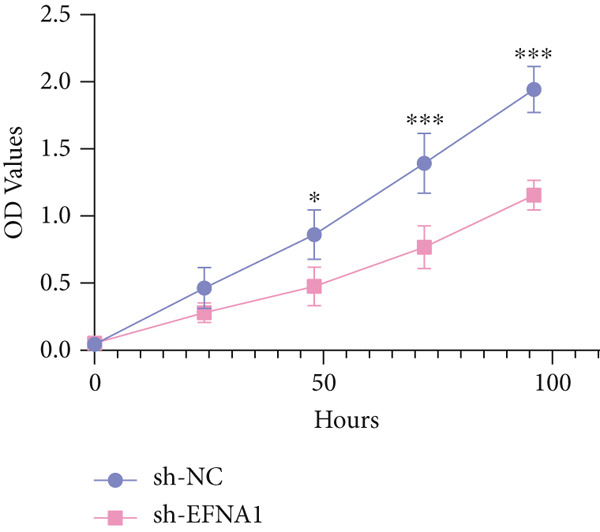
(b)

**Figure:**
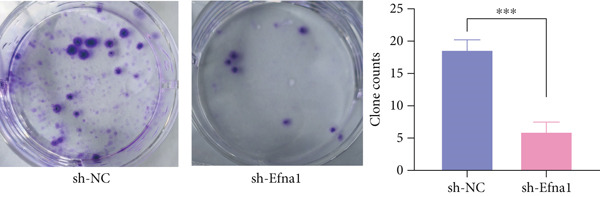
(c)

**Figure:**
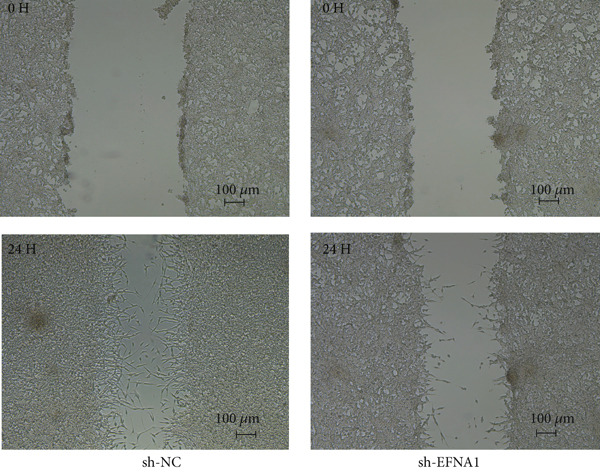
(d)

**Figure:**
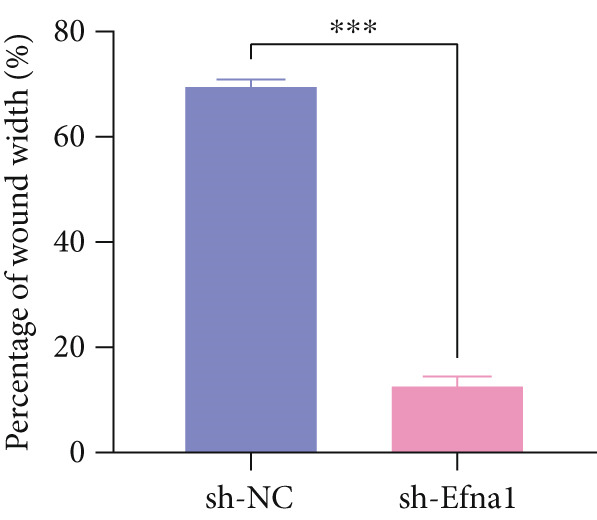
(e)

**Figure:**
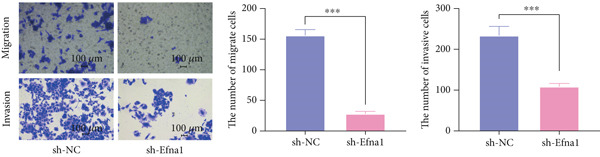
(f)

**Figure:**
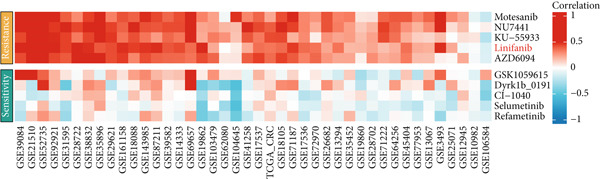
(g)

**Figure:**
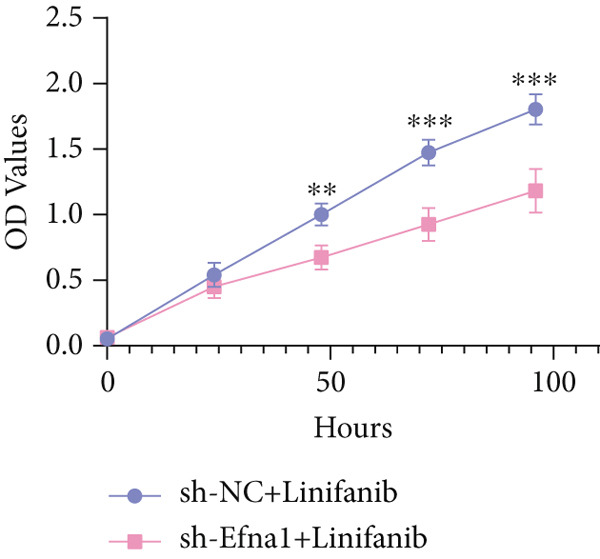
(h)

**Figure:**
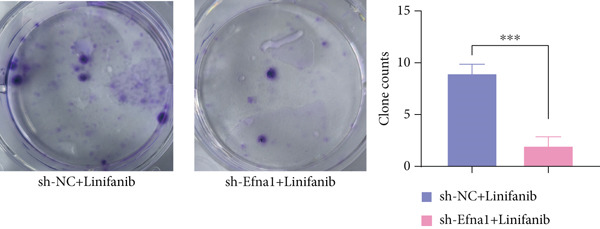
(i)

**Figure:**
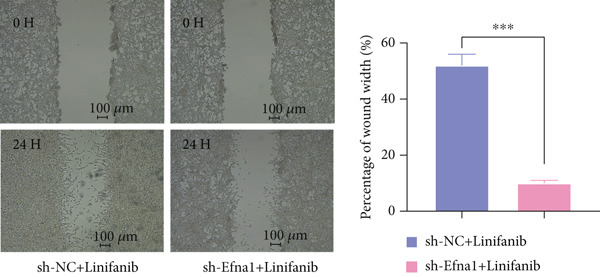
(j)

**Figure:**
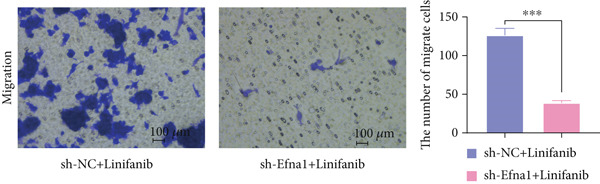
(k)

**Figure:**
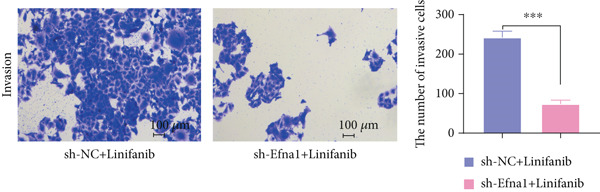
(l)

## 4. Discussion

This study systematically elucidated the cellular and molecular mechanisms of colorectal cancer LNM by integrating bulk RNA sequencing, single‐cell transcriptomics, and computational modeling. By combining phenotype‐associated cell state identification with hdWGCNA and cell–cell communication mapping, we identified a specific MC subpopulation that drives LNM through an IRF9‐mediated transcriptional program and revealed EFNA1 as a key mediator of immunosuppressive microenvironment remodeling. These findings provide a comprehensive molecular framework for understanding colorectal cancer LNM and reveal actionable therapeutic targets within the metastasis‐prone MC state.

In recent years, integrating bulk RNA sequencing and single‐cell sequencing data has become an emerging strategy for constructing cancer prognostic signatures [23]. Traditional bulk sequencing can provide tissue‐level transcriptome information but cannot capture intratumoral cellular heterogeneity, whereas single‐cell sequencing offers high resolution but often lacks complete clinical information annotation. The scPAS method we employed ingeniously bridges the advantages of these two technologies, using phenotype labels from bulk sequencing cohorts to precisely identify cell subpopulations highly associated with LNM in single‐cell data [3]. This integration strategy not only overcomes limitations of single technologies but, more importantly, enables precise localization of truly metastatic‐potential cells in complex tumor samples rather than simply treating all MCs in metastatic samples as a homogeneous population. This multi‐scale integration approach has demonstrated value in constructing prognostic models and identifying therapeutic targets across multiple tumor types [24]. Our study further extends this methodological framework, not only constructing an NRS scoring system with good predictive performance but also deeply exploring transcriptional regulatory networks and cell–cell communication mechanisms driving metastatic phenotypes. Notably, the top 10 high‐coefficient genes we identified (e.g., TSC22D3, VEGFA, APLNR) are highly consistent with known biological characteristics of colorectal cancer metastasis, validating our method′s reliability.

Our study revealed IRF9 as the primary transcriptional regulator of scPAS+ cell phenotype formation. IRF9, a member of the interferon regulatory factor family, has been demonstrated to possess pro‐oncogenic properties in lung cancer, promoting tumor progression and metastasis by regulating versican expression [25]. More importantly, in inflammation‐associated carcinogenesis models of colorectal cancer, IRF9 regulates IL‐6 transcription through direct binding to the IL‐6 gene promoter region, subsequently activating STAT3 signaling to promote tumorigenesis [26]. This is completely consistent with our observations of high IRF9 expression in LNM patients associated with poor prognosis. However, IRF family members exhibit obvious context‐dependent roles in tumors, acting as both tumor suppressors and tumor promoters [27]. This duality may relate to tumor type, microenvironment background, and IRF9 posttranslational modification status. In certain tumors such as triple‐negative breast cancer, tumor‐intrinsic IRF9 expression loss paradoxically predicts increased metastatic recurrence risk and poor chemotherapy response [28]. These seemingly contradictory observations suggest that IRF9 may activate different downstream transcriptional programs in different tumor types. In our study, IRF9 primarily exerts prometastatic functions by activating the MC‐M2 gene module, which is enriched in stress response, cell survival, and migration‐related genes rather than classical Type I interferon response genes, potentially explaining IRF9′s pro‐oncogenic function in colorectal cancer.

Our cell communication analysis revealed that scPAS+ cells establish far more dense and complex communication networks than scPAS− cells. Among numerous communication molecules, EFNA1′s role is particularly prominent. EFNA1, as a ligand for EphA receptors, is highly expressed in various gastrointestinal tumors and closely associated with TNM staging and LNM [13]. EFNA1 participates in tumor cell proliferation, invasion, and metastasis through interactions with EphA2 [29], completely consistent with our in vitro functional experiment results. More importantly, our study systematically revealed the complex association between EFNA1 and the immune microenvironment. Surprisingly, tumors with high EFNA1 expression present a unique immunological profile: low immune checkpoint expression (including PD‐L1), low immune infiltration, yet still achieving immune evasion and promoting metastasis. This pattern contrasts sharply with MSI tumors. MSI‐type colorectal cancers are characterized by high TMB, abundant CD8+ T cell infiltration, and high immune checkpoint expression, responding well to immune checkpoint inhibitor therapy [30]. We observed that high EFNA1 expression mainly occurs in MSS tumors, which typically exhibit low immune checkpoint expression and insufficient immune infiltration [30], representing an immune evasion subtype independent of the classical PD‐1/PD‐L1 axis. EFNA1 may construct immunosuppressive microenvironments by promoting angiogenesis, matrix remodeling, and recruiting immunosuppressive cells [13]. Our cell communication analysis supports this hypothesis: scPAS+ cells not only highly express the VEGFA‐VEGFR2 communication pair promoting neovascularization but also establish close connections with fibroblasts and stromal cells through integrin signaling, systematically remodeling the tumor microenvironment. This multilayered microenvironment remodeling strategy may be more effective and difficult to overcome than simply upregulating immune checkpoint molecules.

Our study found that communications between scPAS+ cells and stromal cells are highly enriched in integrin signaling pathways, particularly LAMB3‐ITGB1 and LAMA3‐ITGA6 ligand‐receptor pairs. Laminin‐332 (encoded by LAMA3, LAMB3, and LAMC2) is an important component of basement membranes, regulating cell adhesion, migration, and invasion through binding to integrin receptors such as *α*3*β*1 and *α*6*β*4 [31]. In breast cancer models, silencing *α*3*β*1 integrin significantly inhibited spontaneous lung metastasis and experimental lung colonization, suggesting *α*3*β*1 integrin plays a key role at metastatic sites [32]. LAMB3 and LAMC2 are upregulated in various gastrointestinal tumors, associated with tumor malignancy and poor prognosis [33]. Integrins not only provide traction force required for tumor cell invasion but also promote cell movement by regulating matrix‐degrading proteinase localization and activity and activating FAK‐Src signaling pathways [34]. Our survival analysis confirmed that patients with high LAMB3 expression had significantly shortened OS, validating the clinical relevance of the integrin‐laminin axis in colorectal cancer metastasis. Importantly, tumor‐derived exosomes can be directed to specific organs through surface integrin expression patterns, participating in premetastatic niche establishment [35], suggesting scPAS+ cells may function not only at primary sites but also remotely regulate lymph node microenvironments through secreted exosomes.

Our study revealed that scPAS+ cells highly express VEGFA and establish strong communication with endothelial cells through VEGFA‐VEGFR2 signaling. VEGF signaling may play dual roles in LNM: promoting tumor neovascularization to provide nutrients and oxygen for tumor growth and ephrin‐A1 regulating VEGF expression through angiogenesis‐dependent mechanisms to promote metastatic dissemination [13]. This VEGF signal activation provides a mechanistic basis for the anti‐angiogenic therapy sensitivity we observed. Linifanib is an oral multitarget receptor tyrosine kinase inhibitor exerting antitumor and anti‐angiogenic effects by selectively inhibiting VEGFR1‐3, PDGFR, and c‐KIT [36]. In a Phase II clinical study of KRAS‐mutated metastatic colorectal cancer, although objective responses were not observed, over 60% of patients achieved disease stabilization, suggesting the drug has certain antitumor activity in such tumors [37]. Among multiple targeted agents screened in the GDSC pharmacogenomic database, Linifanib exhibited the strongest positive correlation between EFNA1 expression and drug resistance profiles. Accordingly, our drug sensitivity analysis revealed that high EFNA1 expression was associated with reduced sensitivity to Linifanib, whereas Efna1 knockdown significantly enhanced colorectal cancer cell responsiveness to this drug. EFNA1‐EphA2 signaling may intersect with VEGF‐mediated angiogenic pathways through reciprocal modulation of endothelial activation and receptor phosphorylation, which could explain the observed enhancement of Linifanib sensitivity upon Efna1 depletion. This finding has important translational significance. Our combined treatment experiments confirmed that Efna1 knockdown combined with Linifanib produced synergistic effects, not only inhibiting cell proliferation and colony formation but also significantly weakening cell migration and invasion capabilities. This suggests that targeting the EFNA1‐EphA2 signaling pathway may be an effective strategy to overcome VEGFR inhibitor resistance. Anti‐angiogenic tyrosine kinase inhibitors have shown clinical benefits in renal cell carcinoma, hepatocellular carcinoma, and colorectal cancer. Although Linifanib failed to demonstrate superiority over standard treatment in Phase III clinical trials for hepatocellular carcinoma, our study provides molecular rationale for optimizing VEGFR inhibitor application in EFNA1 high expression colorectal cancer subpopulations, particularly by combining targeting of the EFNA1 pathway to enhance anti‐angiogenic therapy efficacy, offering new strategies to overcome anti‐angiogenic therapy resistance.

In summary, through integrating multi‐scale transcriptomic data and computational modeling, this study systematically revealed cellular and molecular mechanisms of colorectal cancer LNM. The IRF9‐MC‐M2 axis‐driven scPAS+ cell subpopulation we identified constructs immunosuppressive microenvironments and promotes LNM by remodeling cell communication networks, particularly EFNA1‐mediated signaling pathways. These findings not only deepen our understanding of colorectal cancer metastasis biology but, more importantly, provide new prognostic assessment tools and therapeutic targets for clinical practice. Although IRF9 activity strongly correlates with EFNA1 expression within the MC‐M2 module, direct regulatory binding has not yet been experimentally verified. Future work involving ChIP‐seq or reporter assays will be required to establish causal regulation. The NRS scoring system can be used preoperatively or postoperatively to identify patients at high risk for LNM, guiding adjuvant therapy decisions. EFNA1, as an immune evasion mediator independent of classical immune checkpoints, provides new insights for immunotherapy of MSS colorectal cancer. Targeting the EFNA1‐EphA2 signaling pathway or combining with Linifanib may bring new therapeutic options for metastatic colorectal cancer patients. Preclinical studies have demonstrated that the EFNA1–EphA2 axis can be targeted by small‐molecule inhibitors or monoclonal antibodies to suppress tumor angiogenesis and invasion [38]. Other Ephrin family members, such as EFNB2 and EFNA4, also contribute to tumor progression and metastatic potential, highlighting the conserved prometastatic functions of the Eph–ephrin network. Linifanib, whereas exhibiting potent anti‐angiogenic efficacy, is associated with on‐target toxicities including hypertension and proteinuria, as reported in clinical trials. Future applications may benefit from careful dose optimization, biomarker‐guided patient selection, or combination strategies to minimize adverse effects and enhance therapeutic windows. Future clinical trials are needed to further validate the safety and efficacy of these strategies to improve colorectal cancer patient prognosis. However, our study has several limitations. Future validation in large‐scale prospective cohorts is needed to confirm the clinical utility of the NRS scoring system and EFNA1 as prognostic biomarkers. Additionally, our study primarily focused on LNM, whereas liver metastasis is another important metastatic route in colorectal cancer, with potentially different cellular and molecular mechanisms [39]. Future studies should compare metastasis‐driving cell subpopulations and microenvironment characteristics at different metastatic sites (lymph nodes, liver, lung, etc.) to develop individualized treatment strategies for different metastatic patterns. While our integrative framework combining scPAS, hdWGCNA, SCENIC, and CellChat provides comprehensive multilayered insights, each computational method has inherent assumptions and potential limitations. scPAS depends on accurate phenotype–cell mapping across bulk and single‐cell datasets; hdWGCNA may be influenced by data sparsity and parameter selection; SCENIC is sensitive to transcriptomic depth and motif enrichment thresholds; and CellChat relies on curated ligand–receptor databases that may not capture all context‐specific interactions. These methodological constraints should be considered when interpreting the findings.

## Ethics Statement

All methods were carried out in accordance with relevant guidelines and regulations.

## Disclosure

All authors have reviewed the final version of the manuscript and approved to submit to your journal. All authors have agreed to publish this manuscript.

## Conflicts of Interest

The authors declare no conflicts of interest.

## Author Contributions

Wu Ning, Nan Qiao, and Xin Song conceived and wrote the paper. Wu Ning, Nan Qiao, Lei Zhou, Zongze Li, and Lin Zhang analyzed the materials and drafted the manuscript. Xin Song revised the whole paper. Wu Ning and Nan Qiao contributed equally to this study.

## Funding

No funding was received for this manuscript.

## Data Availability

The transcriptomic and clinical data for all patients were obtained from TCGA and GEO databases.
